# Quantitative systems pharmacology modeling sheds light into the dose response relationship of a trispecific T cell engager in multiple myeloma

**DOI:** 10.1038/s41598-022-14726-5

**Published:** 2022-06-29

**Authors:** R. E. Abrams, K. Pierre, N. El-Murr, E. Seung, L. Wu, E. Luna, R. Mehta, J. Li, K. Larabi, M. Ahmed, V. Pelekanou, Z.-Y. Yang, H. van de Velde, S. K. Stamatelos

**Affiliations:** 1 Sanofi, 55 Corporate Dr, Bridgewater, NJ 08807 USA; 2 Sanofi, 13 quai Jules Guesde 94403 Cedex, VITRY-SUR-SEINE, Vitry/Alfortville, France; 3 Sanofi, 270 Albany St., Cambridge, MA 02139 USA; 4 Sanofi, Orlando, FL USA; 5 Sanofi, 50 Binney St., Cambridge, MA 02142 USA; 6 Present Address: Daichi Sankyo, 211 Mt. Airy Rd., Basking Ridge, NJ 07920 USA; 7 Present Address: Modex Therapeutics, 22 Strathmore Road, Natick, MA 01760 USA; 8 Present Address: Bayer Pharmaceuticals, Cambridge, MA 02142 USA; 9 Present Address: Bayer Pharmaceuticals, PH100 Bayer Boulevard, Whippany, NJ 07981 USA

**Keywords:** Computational models, Myeloma

## Abstract

In relapsed and refractory multiple myeloma (RRMM), there are few treatment options once patients progress from the established standard of care. Several bispecific T-cell engagers (TCE) are in clinical development for multiple myeloma (MM), designed to promote T-cell activation and tumor killing by binding a T-cell receptor and a myeloma target. In this study we employ both computational and experimental tools to investigate how a novel trispecific TCE improves activation, proliferation, and cytolytic activity of T-cells against MM cells. In addition to binding CD3 on T-cells and CD38 on tumor cells, the trispecific binds CD28, which serves as both co-stimulation for T-cell activation and an additional tumor target. We have established a robust rule-based quantitative systems pharmacology (QSP) model trained against T-cell activation, cytotoxicity, and cytokine data, and used it to gain insight into the complex dose response of this drug. We predict that CD3-CD28-CD38 killing capacity increases rapidly in low dose levels, and with higher doses, killing plateaus rather than following the bell-shaped curve typical of bispecific TCEs. We further predict that dose–response curves are driven by the ability of tumor cells to form synapses with activated T-cells. When competition between cells limits tumor engagement with active T-cells, response to therapy may be diminished. We finally suggest a metric related to drug efficacy in our analysis—“effective” receptor occupancy, or the proportion of receptors engaged in synapses. Overall, this study predicts that the CD28 arm on the trispecific antibody improves efficacy, and identifies metrics to inform potency of novel TCEs.

## Introduction

Despite recent advances, MM is not considered curable as most patients will eventually relapse and are likely to develop refractory disease. Once patients develop resistance to established agents, especially to the anti-CD38 mAbs, the survival outcome is dismal. Patients with MM that is refractory to (proteasome inhibitors) PIs, (immunomodulatory drugs) IMiDs or anti-CD38 mAb therapies remain in very serious condition with significant co-morbidities undermining quality of life and resulting in poor overall survival (OS). In a study of 275 heavily pretreated MM patients refractory to an anti-CD38 mAb who have received a median of 4 lines of therapy (range 1–16), it was shown that the median OS from the time of development of anti-CD38 resistance was about 8.6 months. Those who were also refractory to IMiDs and PIs fared worst with median OS of only 5.6 months^1^.

Several approaches focusing on T-cell mediated myeloma cell killing, such as CAR-T-cells^2^ and bispecific T-cell engagers^3^ are currently being investigated as therapy for these patients. In addition, the introduction of anti-CD38 mAbs (ie, daratumumab and isatuximab) has significantly influenced the management of MM. The first generation of anti-CD38 mAbs, daratumumab and isatuximab, has been approved in multiple settings, including single agent (daratumumab) and in combinations (both daratumumab and isatuximab)^4–8^. In most settings, the treatment with daratumumab or isatuximab continues until disease progression which eventually leads to anti-CD38 refractory disease. For these patients, there is a need for novel therapeutic strategies to overcome this resistance.

In this paper, we introduce a trispecific TCE which targets CD38 on MM cells and CD3 on T-cells, similarly to bispecific T-cell engagers, but includes a CD28 arm which can bind to both tumor and T-cell antigens as well^9^. T-cells are mature lymphocytes which can be distinguished by the T-cell receptor (TCR) surface molecules that they express and can be subdivided into many different subtypes.^10,11^ Central and effector memory T-cells are retained after an initial response to an infection dampens, and can be activated to perform effector functions upon antigen re-exposure when their TCR is engaged^12^. Naïve T-cells initially remain in an uncommitted state until their TCR is engaged, and they are activated. Naïve T-cells require co-stimulation to become fully activated, which can be provided through CD28 engagement, making CD28 an good potential therapeutic target^13^. In addition to serving a role in T-cell activation, CD28 antigen is also expressed in multiple myeloma. It appears on primary MM cells in approximately one-third of newly diagnosed patients and it increases in frequency during myeloma progression and correlates with poor prognosis and aggressive features of myeloma^4,14^ By providing co-stimulation to T-cells and additional targets on tumor cells, a potent multi-specific TCE might be able to exert a strong immune response and rescue RRMM patients. This approach can be extended in other formats beyond CD28 such as by engaging two antigen targets on tumor cells to promote tumor-directed specificity.

Due to the complex interactions involved in T-cell engager therapies, and the multiple combinations of receptor levels, cell types, and cell numbers involved, quantitative modeling can be very valuable to better understand the clinical behavior of these drugs^15,16^. PBPK models of bispecific and lymphocyte-targeted monoclonal antibodies have been developed to predict the effect of target binding and lymphocyte movement on the plasma concentration, tumor infiltration, receptor occupancy, and synapse formation generated from these drugs^17^. In addition, PK/PD models have connected bispecific antibody-driven synapse formation to tumor killing through empirical representations of T cell activation or through considerations of active and effector cell numbers^18,19^. Finally, more complex models have been developed, representing T-cell activation, and accounting for regulatory vs. effector T-cell types to predict clinical efficacy and safety of immunotherapy drugs and identify biomarkers of interest^15,20,21^. However, as of yet there have been no modeling studies performed to predict the complexities of the dose–response relationship for trispecific antibodies. Our QSP model of the trispecific antibody aims to understand the specific mechanisms and effects of this drug, and to predict how the design of this novel therapy can provide advantages compared to standard TCEs. Our initial model was extensively calibrated to and validated with in vitro data and can be used to understand dose–response relationships and identify drivers of efficacy across different dose levels.

## Results

### Our QSP model of T-cell engagers utilized literature-derived assumptions about cellular interactions and behavior to form a rule-based model generation code which allowed for efficient and reliable model development

**Figure 1:**
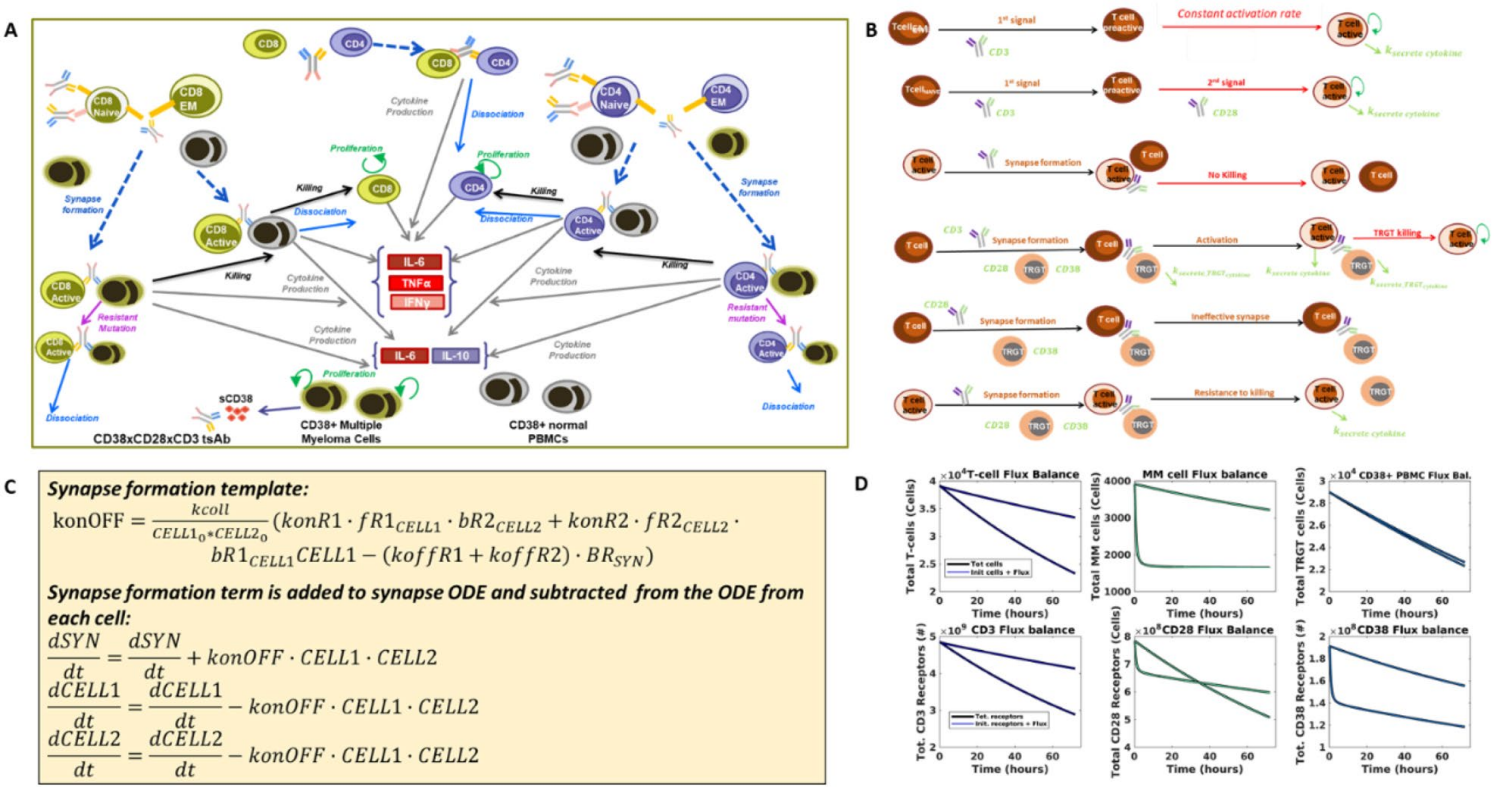
Our trispecific T-cell engager model was designed through a rigorous development process. Here we depict the four key steps in our model development process. (**A**) We first formulated a model diagram showing all the cells and interactions to be included in the model. We included eight key cells—naïve, effector memory, and active T-cells of CD4 or CD8 lineage, multiple myeloma cells, and CD38 + PBMCs. Interactions and processes included in the model are shown with arrows and labeled. (**B**) Assumptions made to determine how interactions should be mathematically formulated in the model are shown. Assumptions were made about the process of activation, synapse formation, killing, and resistance to killing based on literature research and internal discussions (Table S1). (**C**) The model code was generating by encapsulating all cells, synapses, receptors, and interactions in a rule-based model generation code. An example of the template used for the synapse formation term is shown. This term is added to the ODE for the synapse and subtracted from ODEs of the cells joining the synapse. (**D**) We verified that the assumptions and ODEs generated were properly executed by ensuring that flux balances were conserved in the model. The rule-based code produced an ODE for the net flux in each cell and receptor type (i.e. amount added/subtracted over time), which was then added to the initial cell/receptor number (blue lines). We compared this to the total number of cells and receptors across the simulation (black lines) to ensure that all species are conserved. In each panel, results are shown for two doses (8.4e−4 nM and 0.672 nM).

Our model incorporated several key assumptions about T-cell activation and other cellular behavior, illustrated in Fig. 1A,B, and described with references in Table S1. Key among these were the assumption that effector memory T-cells are activated upon CD3 engagement and synapse formation, whereas naïve T-cells required CD28 co-stimulation to become fully activated. We assumed that T-cell-T-cell synapses do not result in killing, whereas PBMCs and tumor cells were killed at different rates. Tumor cells could exhibit resistance to killing, or they could become engaged in ineffective synapses, meaning synapses which do not result in T-cell activation or tumor cell killing. These ineffective synapses include inactive T-cells bound in synapse through the CD28 arm, which cannot lead to T-cell activation or tumor killing, or tumor cells synapsed with other tumor or PBMCs, which again cannot promote tumor killing or T-cell activation.

We developed a model generation code to create a template for the trispecific antibody model which can be easily altered to reflect the presence of different cells and different synapse combinations. The model generation code is a traversal algorithm for all actions and interactions between cells and synapses in the model. By using this approach to create new model equations rather than adding new terms line by line, we achieve a huge improvement in the speed at which new model variants can be built. In addition, we minimize the chance of error, because all new additions to the model follow the same template. At the beginning of the code, cells and receptors are specified. Only synapses involving combinations of receptors specified are generated in the model equations, creating a fit-for-purpose model which is very small and efficient for low-complexity in vitro systems (see procedure overview in Fig. S1).

An example of the rule used to control synapse formation is shown in Fig. 1C. Synapse formation is determined by on and off binding rates of the drug for each receptor, as well as a unitless collision rate, which controls the speed at which cell encounters occur. The synapse formation template can be applied to all cell and receptor combinations initially specified in the code. It is then automatically added to the ODEs for the cells joining the synapse and the resulting synapse. Corresponding equations for the effect of synapse formation on receptors on each joining cell and receptors on each resulting synapse are generated similarly.

The model generation code also allows us to automatically generate flux balance calculations. Each time a cell or receptor is created or destroyed from the model (not including cells/receptors that transfer between states such as free or synapsed), we add this term to a synthesis or degradation flux derivative for that cell/receptor. When we run the model, the overall fluxes are calculated along with the model simulations. We can then compare total cell / receptor number to the initial number + net fluxes each time the model is run, to ensure that flux balance is maintained and to quickly spot any problems in specific cell or receptor types (Fig. 1D).

### Our model calibration process minimized parameter uncertainty by utilizing distinct datasets to inform each important model interaction and produced a well-qualified final model

**Figure 2:**
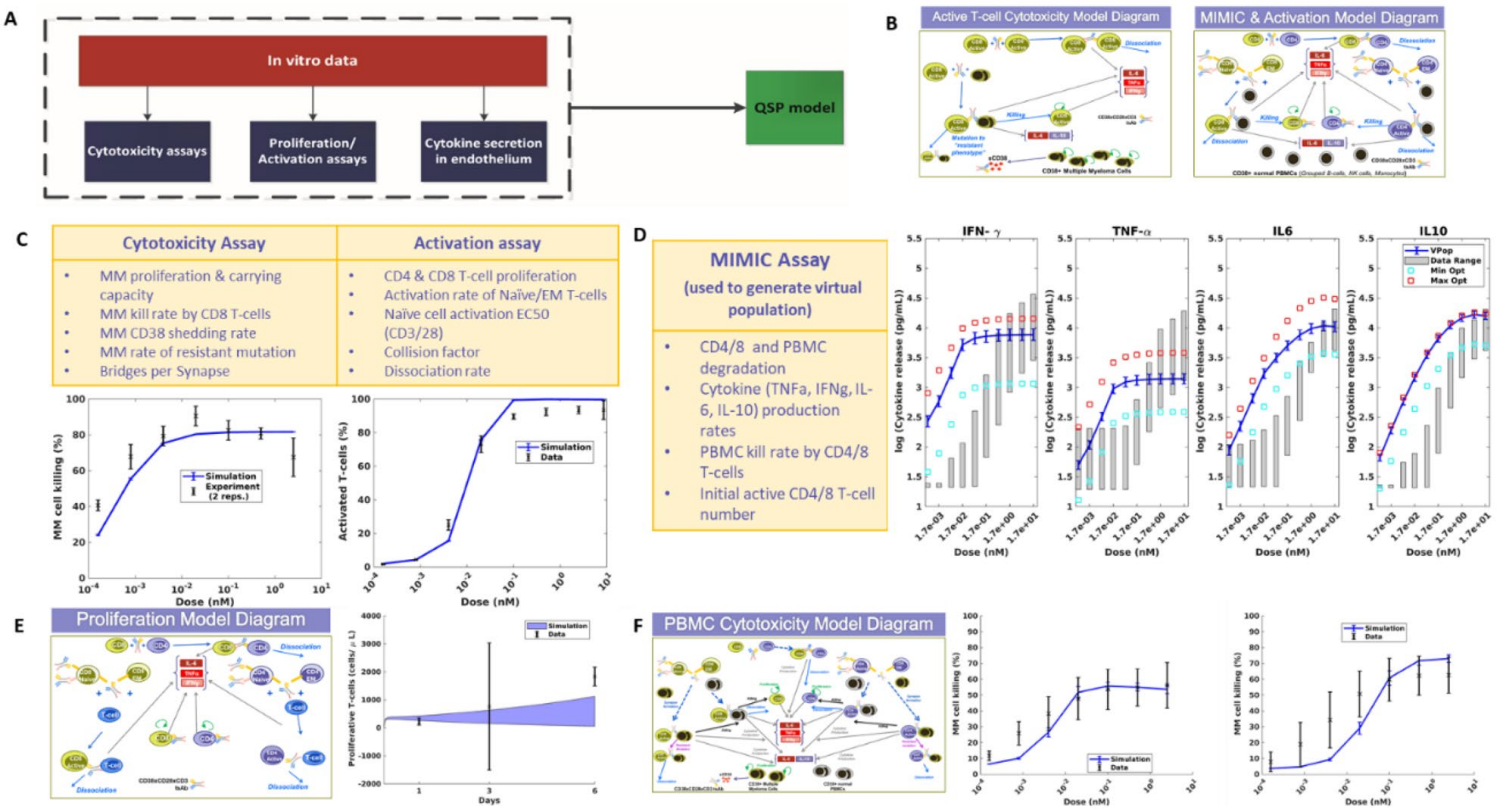
In vitro model training and qualification process ensured accurate prediction of both T-cell activation and tumor cell killing. (**A**) Diagram depicts the overall process of our model calibration, which involved calibration to three separate in vitro datasets, then compilation of all optimized parameters into one QSP model used for analysis and prediction. (**B**) Two model formulations were used for model calibration, designed to replicate the setup of the in vitro experiments performed to generate the data. A model of pre-activated CD8 T-cells and tumor cells was used to train the cytotoxicity parameters to data. A model of PBMCs was used to train T-cell activation and cytotoxicity data. (**C**) Parameters optimized and final model simulation compared to data on cytotoxicity and T-cell activation. (**D**) Parameters optimized to MIMIC assay cytotoxicity measurements. Blue line shows mean + /− SD of population generated from optimization in comparison to range measured experimentally (gray shaded bars). Boxes show optimization results from a traditional distance-based fitting approach to the maximum (red) or minimum (light blue) values of the MIMIC data. (**E**) Model diagram and output generated from tumor proliferation qualification. (**F**) Cytotoxicity model diagram used for qualification of model killing predictions for RPMI-8226 (left) and KMS-11 (right) cell lines.

The model calibration was performed in stages to three in vitro experiments (Fig. 2A). Each experiment demonstrated a different function of the antibody and could be used to inform different key subsets of model parameters and minimize uncertainty in these parameters. Two different model structures were used for optimization, to match the cells included and interactions represented in the experimental setups (Fig. 2B). We first optimized T-cell activation parameters because the activation process is the key determinant of drug potency and the potential for effective synapse formation. All parameters related to T-cell activation and synapse formation were optimized to data on the percentage of activated T-cells in PBMCs. Because there were no MM cells in this incubation, this allowed fitting of the key activation parameters without significant cell killing taking place (Fig. 2C). We next optimized the killing of MM cells. The experiments used incubated pre-activated CD8 T-cells with two MM cell lines, so the number of species and parameters involved in this version of the model were very limited and well-informed by the data. Activation rates were not relevant because the cells were pre-activated, but the parameters controlling synapse formation which were previously optimized did inform the simulation and optimization of tumor killing. A resistance mechanism for tumor cells was added to the model to account for the maximum efficacy of the drug of less than 100%. Cytotoxicity data was calibrated for both the CD38 high (1.29 × 10^5^ receptors/cell) RPMI cell line (Fig. 2C) and the CD38 low (2.5 × 10^3^ receptors/cell) KMS-11 cell line (Fig. S4). Data used for these two calibrations is shown in Fig. S2–3.

We finally used the MIMIC assay to calibrate predictions of cytokine emission in the model, and to establish an in vitro population to use our simulations. The results from this experiment showed a wide range of cytokine levels produced (Fig. S5), and so in order to capture the full range of cytokine responses possible, we calibrated our model to fit the range of this data, rather than the median value (Fig. 2D). To check the validity of the parameter ranges identified by this method, we also ran separate optimizations to establish maximum and minimum parameter values and found that the population predictions fell within the limits generated by a traditional optimization method.

To qualify the validity of our approach, we used two separate in vitro experiments. One experiment measured the concentration of proliferative T-cells over a 6-day time course, following incubation of primary human T-cells with a high concentration of the trispecific antibody, to give us confidence in our predictions of T-cell activation. The other experiment was chosen to validate our predictions of cytotoxicity of the drug. In this experiment, tumor cells were incubated with a population of PBMCs, so that both T-cell activation and cytotoxicity parameters were important to ensure that the data was accurately predicted. Both simulations required different model structures, shown in Fig. 2E,F. To run simulations of these experiments, we used our in vitro population, and altered the types and initial numbers of cells in the simulation to match the experimental setup of 10 PBMCs: 1 tumor cell. We show in Fig. 2E,F that our model was able to accurately predict both T-cell activation over a 6-day time course, and MM cell killing across different doses for two different tumor cell lines. Data used for Fig. 2E is shown in Fig. S6, while data for Fig. 2F was published in^9^.

### Model sensitivity analysis reveals that while increasing the synapse number is most important to promote killing at low doses of drug, the types of synapses formed are most important at high doses

**Figure 3:**
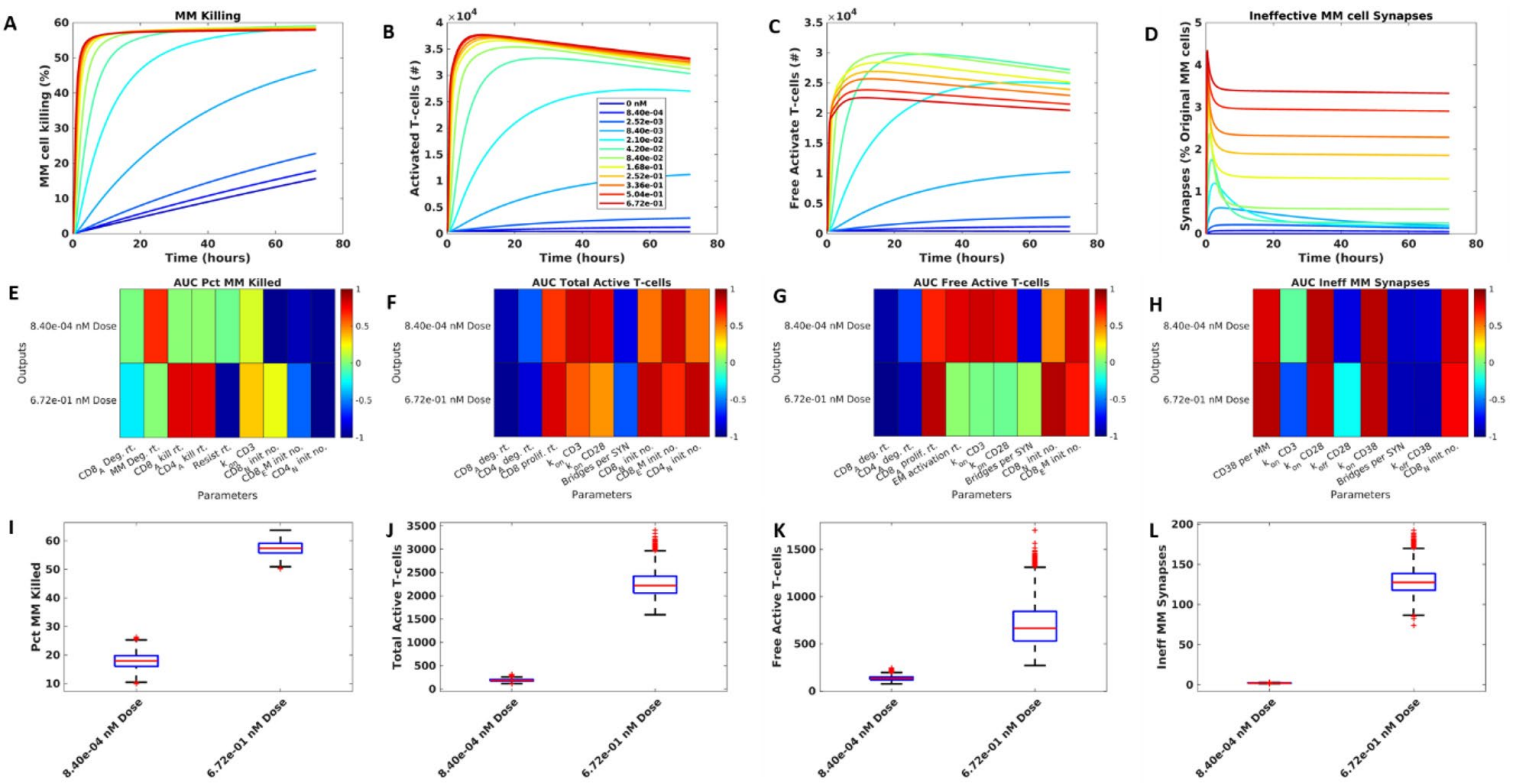
Efficacy at low doses increases with increasing synapse number while high dose level efficacy is driven by decreasing the number of ineffective synapses through better access to active T-cells. One set of values from the final population was selected and run for three days for different doses of interest. The results for tumor killing (**A**), Total active T-cells (**B**), Free active T-cells (**C**), and ineffective T-MM synapses (**D**). The sensitivity of these outputs to parameters is plotted as an AUC heatmap, where dark red = positive correlation, dark blue = negative correlation (**E–H**). Sensitivity is shown at two doses, indicated on plots. Parameters shown in each heatmap have 80% or more correlation to least one evaluation metric (such as AUC) derived from the corresponding output. Variability of each output across doses in the sensitivity analysis population (**I–L**).

We performed a PRCC global sensitivity analysis to further examine the forces driving T-cell activation and tumor killing in the model for low (8.4 × 10^–4^ nM) and high doses (6.7 × 10^–1^ nM) of drug. By examining the kinetics of tumor killing, T-cell activation, and ineffective synapse formation over time, we see that lower drug doses lead to slower kinetics for all of these process, whereas high doses lead to a quick steady state (Fig. 3A–D). Killing, total activated T-cell number increase with dose up to a 0.3 nM dose, where they remain steady (Fig. 3A,B). The number of ineffective synapses also increases with dose, without reaching a maximum. However, the level of free active T-cells has a more complex dose–response relationship, increasing to a maximum around 8.4e−2 nM.

We ran a PRCC analysis which produced outputs of interest at two dose levels to study parameter sensitivity. Across the two simulations, killing was increased from 20 to 55% on average (Fig. 3E). Total and free active T-cells and ineffective synapses are close to zero at the lower dose, with little variability, but gain in value and spread for the higher dose (Fig. 3F–H).

To analyze the sensitive parameters of the model, we identified the parameters with higher than 80% absolute correlation to the outputs of interest, (Fig. 3I–L). For tumor killing, we found that for low doses, the binding rate of CD3 receptors was more important for driving killing, because of the need to promote T-cell activation. Killing is so low at low dose levels, that a change in MM cell degradation can be more important in reducing the final MM cell number than the killing itself. For higher doses, the killing rates and resistance rates became much more important in determining efficacy. Across both doses, inactive cells tended to impede killing by competing with active cells for engagement with tumors (Fig. 3I).

The key parameters driving total T-cell activation were straightforward (Fig. 3J), with lifecycle kinetics and pre-active cell numbers most central. The rate of binding to both CD3 and CD28 was highly important at the lower dose, where there is a need to form any synapses. However, at the higher dose, these factors become less important, particularly CD28 binding rate which can also lead to formation of ineffective synapses. The results also show that EM cells are more important for low doses, where amount of drug available to provide co-stimulation is low, and naïve cells are more important at high doses where they can be easily activated.

Free active T-cells showed more complex determinants (Fig. 3K): At low doses, the number of synapses allowing T-cell activation is most important for increasing free active T-cell numbers, with binding rates to CD3 and CD28, activation rates, and initial inactive T-cell numbers all having a strong positive correlation to the free active T-cell number predicted by the model. At high doses, more synapses mean fewer unbound active T-cells, so many parameters listed before have a neutral effect on this output. At the higher dose, having more active cells, through increased proliferation rates and initial pre-active cell numbers, is most important.

We finally looked at how model parameters alter the percentage of MM cells engaged in ineffective synapses, which prevent tumor killing by binding tumor cells to partners which are unable to kill. Across both doses examined in the sensitivity analysis, parameters promoting synapse formation also increased the number of ineffective synapses, such as the number of CD38 molecules per MM cell, and the binding rate to CD28 and CD38 (Fig. 3H). The off rate of CD38 was negatively correlated to ineffective synapse number at low doses, where MM cells escaping these synapses can find a new effective binding partner, but a neutral effect at high doses where most cells are engaged and unavailable to bind. For higher doses, the CD3-binding rate was negatively correlated to ineffective synapse number because it allowed more effective T-cell activating bridges to form. 

The affinity of T-cell engager antibodies for their targets is critical to their activity with optimized-affinity candidates selected based on their potential to elicit significant cytotoxicity without compromising safety^9,30^. Our sensitivity analysis reveals that the drug’s binding kinetic parameters, especially rates relating to CD3 and CD28 antigens, impact outputs of interest. Particularly, both the kon and koff rates for CD28 (where koff = KD x kon) significantly affect the number of ineffective synapses formed at both high and low doses, indicating that the choice of CD28 affinity will dictate the dose–response curve for trispecific antibodies.

### Trispecific effective activation of the T-cell population through co-stimulation of CD28 leads to stable effective receptor occupancy across doses and a strong advantage to trispecific antibody efficacy at lower doses

**Figure 4:**
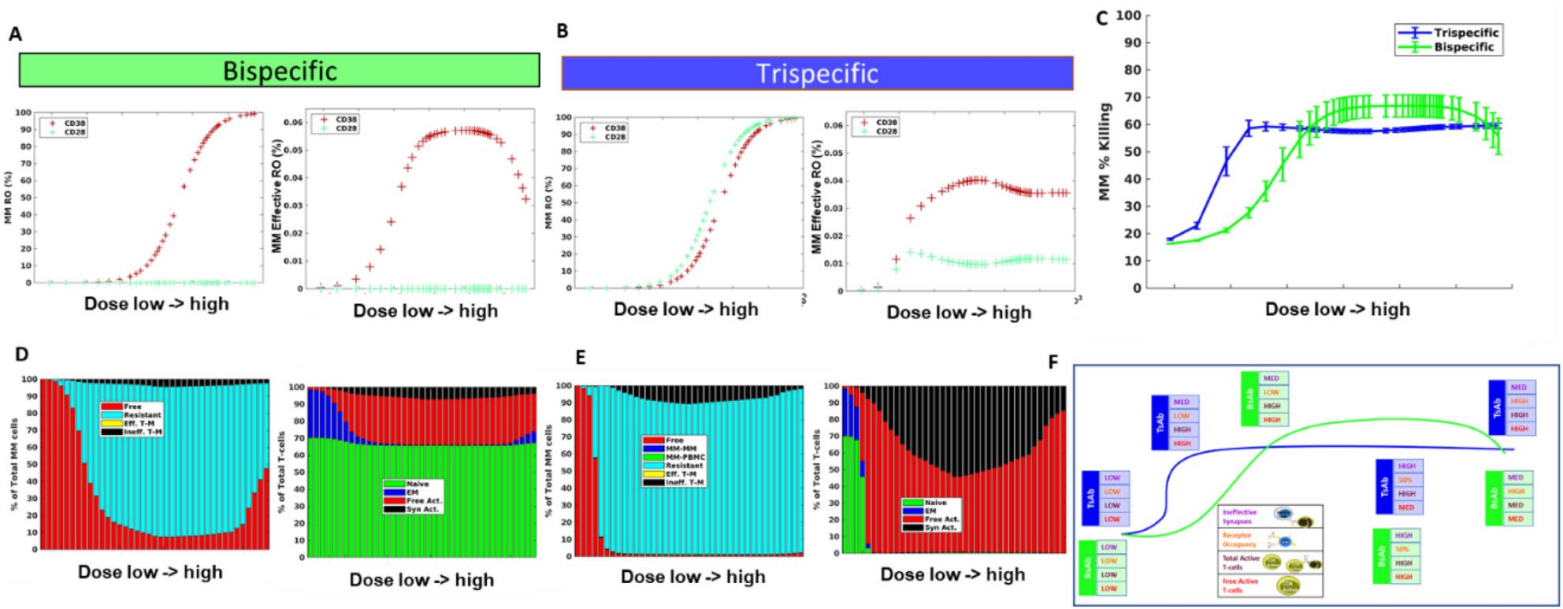
T-cell engager efficacy is driven by several factors leading to highly superior efficacy of trispecific antibodies at lower doses but comparable performance as dose increases. Comparison of trispecific simulated over three days to comparable bispecific with no CD28 binding. MM cell receptor occupancy and “effective” receptor occupancy for bispecific and trispecific, respectively (**A, B**). (**C**) Killing was predicted across the in vitro population for varying doses of the bispecific compared to trispecific. MM cell distribution and T-cell distribution are shown at final timepoint (**D, E**) for bispecific and trispecific, respectively. (**F**) Schematic of the prevalent forces driving dose–response curve. Blue line and boxes describe trispecific dose–response and levels of ineffective synapses, receptor occupancy, total T-cells, and active T-cells. Green line and boxes show bispecific simulation.

In our final analysis, we compared predictions for our trispecific antibody to an analogous bispecific antibody, without the CD28 binding arm, to see if the inclusion of the CD28 binding arm does offer an advantage for our drug. We first compared receptor occupancy for the two drugs (Fig. 4A,B). Across the wide range of doses tested, nearly 100% receptor occupancy (RO) is reached for both the bispecific and trispecific simulations. Note that RO of CD28 for the bispecific antibody simulation remains at 0 because the simulated bispecific was unable to bind CD28. We then calculated an effective RO, to see how this binding translated to the ability of cells to form synapses and engage in bridges to receptors on other cells. Effective RO was defined to be the number of bridges formed for each specific receptor out of that total receptor number (specific to each cell-type), so it gives us a picture of which receptors are actually engaged in intercellular connections by drug. For tumor cells in the bispecific antibody simulations, the effective RO follows the bell shaped curve typical described in PK/PD models of these antibodies^18,22^ (Fig. 4A), where, effective RO increases to a maximum at a level similar to the EC50 RO of CD38 as measured in the typical way, then starts to decline as typical RO increases to full occupancy. However, for the MM cell effective RO predicted by the trispecific antibody there is a notable change (Fig. 4B). The effective RO increases to a maximum value at a lower dose, when RO of MM cells is still low, and it remains relatively stable throughout the doses tested. When we look at the comparison of these effective RO curves to tumor cell killing (Fig. 4C), we find that the effective RO is a good general predictor of the dose–response curve.

A comparison of the killing predicted for bi- and trispecific antibodies shows that the trispecific antibody consistently outperformed or matched the bispecific antibody across all doses tested (Fig. 4C). The largest difference was in the low doses when the trispecific antibody killing was up to threefold increase from the bispecific antibody. In order to better understand what is driving the tortuous dose–response curves predicted for both antibodies, we further examined the distribution of T-cells and MM cells, knowing from the sensitivity analysis that T-cell activation and cell competition could be important for drug efficacy. We found that a significant portion of the tumor cells in the bispecific antibody simulations remained free across all doses, and unable to effectively engage (Fig. 4D). In addition, a small portion of tumor cells were engaged in ineffective synapses, preventing these cells from being killed. An examination of T-cell status for the bispecific antibody case shows that where killing increases, the proportion of T-cells becoming active increases with dose as well; most of these active T-cells remain free and able to bind tumor and execute killing (Fig. 4D). At high doses, killing begins to decrease because a substantial portion of T-cells are not activated, leaving an insufficient number to compete for effective synapses with the antibody. The trends seen in killing, free tumor cells, and amount of active vs. EM T-cells for the bispecific follow the receptor-occupancy driven bell curve^23^ . Note that throughout the simulations, there is a large portion of naïve T-cells which remain inactive due to the inability of the bispecific drug to co-stimulate these cells, and these cells can engage in ineffective synapse formation.

For the trispecific antibody, we see that starting from a low dose, there is a very small portion of unengaged tumor cells, reflecting the ability of tumor to join synapses through both CD28 and CD3 (Fig. 4E). Killing reaches a maximum after a few doses, where the number of free MM cells reach close to zero and most T-cells have become activated. around the point where killing is at a maximum (Fig. 4E). Killing decreases slightly as more active T-cells are bound in synapse, but tumor cells are engaged at such a high level with active T-cells that the effect is minimal. At higher drug doses, we do see some increased ineffective T-cells for trispecific compared to the bispecific, which leads to slightly higher bispecific killing for these doses.

In performing this analysis, it is clear that many factors in the system can alter the predicted outcome of T-cell engager therapies and that it is important to take account not only the receptor occupancy of the drug, but the types of cells, interactions of cells, and number of cells in the system. Our schematic (Fig. 4F) summarizes the key factors controlling outcome in this system that will help predict efficacy. Receptor occupancy, as previously identified, is important in determining the level at which maximum synapse formation will occur, and this determines the minimum killing level for the bispecific antibody. The amount of total and free active T-cells is also an important driver of tumor killing. As levels of free active T-cells fluctuate, tumor killing patterns may follow. Finally, ineffective synapses are important determinants of drug efficacy and are a marker of the ability of tumor cells to compete in the simulated environment. As the number of synapses increase after drug is first administered, the number of ineffective synapses will naturally increase as well. However, the key to reaching maximum efficacy is a drug’s ability to begin to decrease the number of ineffective synapses which is indicative of tumor cells' ability to compete and be killed, and to have a large pool of activated T-cells which can execute the killing. We outline the differences between bi- and trispecific antibodies at each stage and find that the trispecific antibody is able to maintain a large pool of free active T-cells throughout all but the smallest doses tested, enabling it to promote steady tumor killing across doses. Killing is a combined outcome of the ability of the drug to form synapses at low doses (measured by effective RO) and the ability of these synapses to activate T-cells and engage and kill tumor (measured by free and total active T-cells and ineffective synapse number), and all of these factors must be considered in determining final efficacy of the T-cell engager.

We have also investigated the downregulation of MM cells and T cells in patients treated for multiple myeloma to understand how dose–response of the antibody is altered in real life situations (Fig. S8 and S9). The results indicate that the expression differences are not sufficient to change the responses presented in Fig. 4C.

## Discussion

We have developed a QSP model of T-cell engager therapy which is founded in well-supported assumptions based on biological knowledge and data. Our model was calibrated to and validated against several types of experimental setups, with different cell types, receptor numbers, and interactions involved. QSP models are complex, and therefore adjusting the model structure to include several cell types can be time consuming and require intensive error checking. With our model generating code, this process was extremely simple, reproducible, and an accuracy check was built in to ensure flux balance was maintained. This type of multiscale model development can be particularly useful when attempting to utilize high throughput data to understand tumor heterogeneity in the individual patient level which can be incorporated in the model (Zhang et al*.* 2021, Lazarou et al*.* 2020). Our model generation code allows for adaptation of the model building blocks to different tumor microenvironments with various cellular and molecular components that are necessary to describe the MoA of the drug.

It is worth mentioning that software utilize rule-based model-building to facilitate code development: Simbiology (Mathworks, Natick MA) features a common set of kinetic laws which can be populated with different parameter and species names and associated values in the model to simplify model construction. Additionally, software BioNetGen has been developed specifically on the principles of rule-based model design and has been applied in systems biology models of cell-signaling and other systems^24^. Our model utilizes the same principles implementing a sorting algorithm that identifies high commonality among species of the system and ranks them appropriately. Specifically, we employ a standard parameter definition and rule template for processes like proliferation and degradation, where each cell and parameter name must be defined separately. Further, for synapse-level interactions, model construction requires a list of cell pairings and associated receptors. The algorithm traverses the available species and parameters to identify which parameters to use (i.e., find which synthesis rate is appropriate based on the receptor name), and to determine which ODE equations these terms should be added to. This method vastly simplified and sped up our code development and is generalizable to any T cell engager or ADCC system.

Our model examines the drivers of dose–response of T-cell engager therapies, and particularly, trispecific antibodies with common antigens among effector and target cells. Bispecific antibody models have proposed that a bell-shaped dose response curve should be expected, based on the ability of drug trimers to form between cells as receptor occupancy increases on both cell partners^25^. When aligning model predictions with preclinical and clinical responses, trimer concentration has been found not to adequately describe dose–response and additional constructs such as active T-cell numbers, or T-cell potency, have been linked to cytotoxicity to better explain the data^26^. Furthermore, several studies and reviews discuss the complexity of dose–response found for bispecific antibodies in the clinic and the differential success of this drug in patients with different levels of disease^27^. Our model attempts to extend the understanding of dose–response to trispecific T-cell engagers, and to generate a consolidated perspective of what controls dose–response in these modalities. We find that at low doses, T-cell activation and increasing synapse formation controls efficacy and promotes an increasing dose–response over time. As doses increase at the point of maximal T-cell activation, cell competition, ineffective synapse formation, and availability of free active T-cells for binding are most important in controlling dose–response. These factors can lead to complex dose–response curves which vary based on E/T ratio and antigen expression, among other factors. We do see a bell-shaped curve in the number of synapses formed based on receptor occupancy as suggested in^23^, but this often is not translated into effective receptor occupancy (synapse formation) or killing due to the complex landscape of other factors contributing to the overall effect.

Despite their complicated dose–response, bispecific antibodies have had success, and are a continued focus in drug development over recent years^28,29^. Bispecific T-cell engagers have been effective at promoting T-cell activation and killing, but they do not activate all T-cells. They are more effective at activating previously primed T-cells which do not require co-stimulation^30,31^. Note that we do not model the role of antigen-presenting cells in this system, or cytokine feedback regulating the immune response^32^, but we believe that the interactions we represent provide a fundamental foundation for elucidating the main drivers of response. The trispecific antibody binds to CD28 receptor on T-cells and on the MM cells expressing CD28 and thus provides co-stimulation to all T-cells, potentially activating a wider pool of T-cells to kill the tumor^33,34^. While other clinical trials are pursuing the combination therapy of CD3-directed bispecifics with CD28-directed bispecifics^13^, our compound combines these targets in one coherent system. In comparing simulations of the trispecific with a comparable bispecific molecule without CD28 targeting, we find that CD28 does give significant benefit at lower doses. This finding may be more relevant to the clinical active dose than the predictions for higher doses, as many T-cell engaging drugs show high potency at low doses^27^. Furthermore, CD28 engagement keeps a higher proportion of T cells activated for longer period without necessarily leading to exhaustion^34,35^. Finally, the dose–response plateauing potentially indicates that reaching dose liming toxicity may not be the most relevant determinant in identifying the suggested active dose, lower doses may be more important.

An additional insight from our model is that ineffective synapses are more likely to form because T-cells must be engaged in synapse through CD3 in order to become activated^36–39^. While an ineffective synapse is a concept unique to T cell engagers, where cells are actively driven to find a partner, the concept of needing the right type and localization of immune cells to drive therapeutic efficacy is not novel. In fact, the complexity of the immune system and the different pro and anti-inflammatory immune cell types which are part of the immune landscape of an individual patient, were often found to be a key contributor to patient response to therapy. Patients treated with checkpoint immunotherapy have been shown to have exhausted T-cell populations enriched in non-responsive lesions and active T-cells enriched in responsive lesions^40^. It has also been found that tumors can resist immunotherapy by recruiting immunosuppressive cells^41,42^ and promoting T-cell dysfunction, or by excluding T-cells from infiltrating the tumor^43^. Furthermore, MM patients treated with anti-CD38 mAbs can develop resistance through reduced NK cells and reduced CD38 expression on NK cells, impairing the mechanism of action of these drugs by reducing the functionality of the effector cells^44,45^. Our model suggests that, despite ineffective synapse formation, the trispecific antibody can overcome many of these limitations by engaging a larger spectrum of T-cells and keeping them engaged through direct stimulation. Our model has helped to elucidate key cell types and interactions that can determine drug efficacy, and we believe QSP modeling has a critical role to play in identifying the dose and biomarkers of response to guide clinical trial design and advance cancer therapy. Our model has currently informed design of a FIH trial for this therapeutic (ClinicalTrials.gov Identifier: NCT04401020), and appropriately restructured, scaled, and parameterized to predict efficacy dose responses in an in silico clinical trial. Results from this trial will further improve our model design and predictions, allowing us to better understand the activities of this T cell engager as well as other comparable therapies.

## Materials and methods

A flowchart highlighting the primary steps involved in the model development and assessment phases is depicted in Fig. S7.

### Model overview

We have developed a mechanistic model describing the impact of the CD3-CD28-CD38 antibody in the context of multiple myeloma disease. The in vitro model of this drug encompasses cells, receptors, synapses, bridges, soluble CD38 (sCD38), and critical cytokines (Fig. 1). It provides an elaborate representation of the kinetics and dynamics of the phenomenon describing T cell activation and proliferation, MM cell and PBMC killing, resistance to killing and cytokine production. It includes six T-cell subtypes, including CD4 and CD8 T-cells, of the naïve, effector memory, and active type. It also includes multiple myeloma (MM) cells, and a lumped species representing all CD38 + PBMCs, such as B-cells, NK cells and monocytes.

The key interaction represented in this system is the formation of synapses between cells connected by the CD38xCD28xCD3 trispecific antibody (see overview schematic in Fig. 2). Synapses can be formed between any pairwise combination of cells bound to the CD3, CD28, or CD38 arm of the drug. T-cells express CD3 and CD28 antigens, MM cells express CD38 and CD28 antigens, and the model assumes that CD38 + PBMCs only express CD38. Though CD38 is expressed on T-cells and upregulated in active T-cells, the level of CD38 expression is lower than it is on PBMCs, and lower than expression of CD28 on T-cells^46,47^ . Because of this, we decided not to represent CD38 on T-cells in the model because it would likely not add meaningful predictive capability to the model, and would significantly increase model complexity. Synapses formed between T-cells or MM cells do not result in cell killing, while synapses between an active T-cell and a MM cell or PBMC do result in killing at different rates. Some portion of T-cell-MM cell synapses are assumed to become resistant to killing through unspecified mechanisms (e.g. exhaustion), allowing MM cells to escape. The model assumes that sCD38 is secreted by MM cells and can bind to drug leading to drug clearance.

The model represents T cell activation. It assumes that effector memory T-cells can be activated when they are involved in a synapse through their CD3 arm^38,39^, whereas naïve T-cells require co-stimulation of CD28 and CD3 drug binding, and their rate of activation increases as more drug is bound. Active CD8 and CD4 T-cells are both capable of killing, but the model assumes that CD8 cells are more efficient at inducing MM cytotoxicity^48,49^.

The model finally represents cytokine production by T-cells and PBMCs. In the model, active T-cells produce TNF-α, IFN-γ, and IL-6 cytokines. There is no process of PBMC activation included, in order to reduce model complexity, so CD38 + PBMCs produce IL-6 and IL-10 cytokines only when in synapse^50^ . In the current model, cytokines are an output of the model only, cytokine feedback on cell proliferation or cytokine production rates is not represented.

Owing to the mechanistic representation of MM biology, the described complex molecular and cellular crosstalk amongst various immune cells with MM cells, and the versatility of the rule-based model generation script, this QSP model can be extended to characterize the activities of other immuno-oncological MM therapies. For instance, by updating drug binding parameters, effector cell types and corresponding antigen expression levels, and by accounting for cell-independent, antibody-driven MM killing, this in vitro model can describe the activities of other CD38 targeting antibodies such as isatuximab (Zhu et al. 2020) and daratumumab (Michel de Weers et al. 2011).

### Model equations

The full in vitro model used for dose-prediction (Fig. 1) has 760 species and 114 parameters. All cell behavior in the model is described by zero or first-order reactions except for synapse formation and T-cell activation. There is no formal PK model in the in vitro model; a constant level of drug is injected into the system and it is only cleared through PBMC or MM cell killing or synapse dissociation. The model assumes that cytokines are not significantly degraded in vitro. It assumes that sCD38 is not present initially but accumulates through shedding of the soluble receptor from MM cells.

Synapse formation is driven by bridge formation between receptors (Equations shown in Fig. 1C). Bridges can form in any order—drugs first bind to receptor on one cell then bridge to another free receptor using typical kon/koff formulation. Because receptors are on different cells, synapse formation is also driven by the number of cells of each type forming the synapses. We multiply the equation by a nondimensional “collision factor” which is calibrated to data and can be scaled by experiment volume so that cells in more compact spaces are more likely to collide. We also calibrate a “bridges per synapse factor” which describes the number of paired molecular interactions leading to the formation of a cytolytic synapse between two cells^51^. This factor, with a value set to be relatively low, controls how many receptors from each cell are considered to be bound in synapse and unable to bind or unbind to drug.

T-cell activation differs for EM and naïve T-cells. We assume that EM cells can be easily activated without co-stimulation, as it has been shown that these are likely the cell type engaged most by bispecific antibodies^52–55^ EM cells must be bound in synapse through the CD3 arm to become activated, but once this occurs, they transition at a constant rate into active synapses. Naïve cells are likely not effectively engaged in bispecific antibodies because they require co-stimulation to be activated. To this end, naïve cells must have drug bound to CD3 and CD28 to be activated, in addition to being engaged in a synapse through the CD3 arm. We model this transition as an “AND” gate formulation^56^, with Michaelis–Menten terms for bound-CD3 and bound-CD28 multiplied together and by a constant activation rate to control the transition rate from inactive to active cells. If no CD28 is bound with drug, naïve cells are not activated.

### Model generation code

Model generation code was developed in house. We used Matlab 2020a (The MathWorks, Inc., Natick, Massachusetts) with the pattern matching toolbox. Our ability to use pattern matching so successfully was reliant on clear and consistent naming standards throughout the model, so that cell names and receptor names could be easily identified in each species. A copy of the model generation code used to generate the full in vitro model is included as supplementary Matlab files.

### Model calibration

The model calibration was performed in stages to three in vitro experiments (Schematic in Fig. 2A). In all cases, parameter ranges used to set optimization bounds were obtained from the literature, and the genetic algorithm in Matlab 2020a (The MathWorks, Inc., Natick, Massachusetts), were used to run the calibration. Experimental data and methods used to perform experiments are located in supplementary material.

### Model parameters

To obtain well known parameter and initial values, we made use of extensive information from the literature and from internal studies (Table S2). For example, trispecific antibody binding properties and multiple myeloma cell line antigen expression were quantified internally whereas primary cell expression levels of CD3^57^, CD28^47^, CD38^58–61^ were obtained from literature. While experimental conditions determined the drug frequencies and many initial cell numbers, our model still often required further literature research to define initial species. Experiments were often performed by incubating with PBMCs, or with isolated T-cells, so we obtained estimates of blood immune cell subtypes^62^ and CD4 + /CD8 + T cell subset distributions^35^ to use in further delineating how many cells of each type should be defined.

Moreover, the dose prediction module of the in vitro model was not based on a specific experiment, but was designed to represent a sample of peripheral blood from a human multiple myeloma patient, including T-cells, MM cells, and PBMCs at proportions relevant to numbers of immune cells and circulating tumor cells in human peripheral blood subtypes^35,62^.

### Model simulation

The final in vitro model used optimized values from the activation and cytotoxicity calibrations, and optimized population values from the MIMIC calibration to make a virtual population. For qualification experiments, cell numbers and simulation times specified for each experiment were used to run the model (6 days incubation with 1 × 10^5^ sorted T-cells for the proliferation experiment, 24 h incubation with 2 × 10^5^ PBMCs and 2 × 10^4^ MM cells for the cytotoxicity experiment). Optimized EM and active cell numbers from the MIMIC experiment were not used absolutely to initialize the in vitro model, but were scaled to total T-cell number, so that the ratio of initial cells was maintained across experiments. For the simulations, initial cell numbers were based on literature findings for the peripheral blood of an MM patient, as described in more detail in the parameters section. Time of simulation was 24 h for initial cytotoxicity and killing predictions (to be comparable to the MIMIC assay setup), and three days in later simulations, to allow simulations to reach more of a steady state. The dose prediction simulation tested dose levels that started at the MABEL dose and spanned the range of interest for the human doses.

The bispecific antibody was simulated by setting kon for CD28 to 0, so that drug could only bind to CD38 and CD3. No other specific changes were made, but this alteration in binding eliminated the possibility of T-cell-T-cell synapses, MM-MM synapses, or MM-PBMC synapses, because there was no second antigen to bind. This change also altered the classification of ineffective or effective synapses. We define an effective synapse to be one in which an MM cell is joined that can lead to either T-cell activation or killing. For the trispecific antibody, MM cells in synapse with Naïve or EM T-cells may be considered effective if they are bound through CD3 on the T-cell, and both CD3 and CD28 have some receptor occupancy on these cells. MM cells bound to other MM’s or PBMCs are also ineffective. In the bispecific simulation, naïve cells cannot be activated at all because there is no co-stimulation present, so these are ineffective synapses.

All simulations were run in Matlab v2019a or 2020a, using a CVODE wrapper^63^ to improve runtime.

### Global sensitivity analysis

We performed a global sensitivity analysis using the PRCC method (^64^, Code from: Simeone Marino, May 29, 2007). All parameters were varied by ± 10% from the final model values. The PRCC used the same model setup as the dose simulations (i.e., 24-h runtime, cell composition meant to represent human peripheral blood). We ran the GSA on two different dose levels, low and high, and calculated the AUC for each output of interest to use in performing the sensitivity analysis. Parameters with a significance of better than 0.01 on outputs of interest were retained for the analysis.

## Supplementary Information

Supplementary Information 1.

Supplementary Information 2.

Supplementary Information 3.

Supplementary Information 4.

## Data Availability

The datasets generated during and/or analyzed during the current study are available from the corresponding author on reasonable request.
