# Supplementary material for: Quantitative systems pharmacology modeling sheds light into the dose response relationship of a trispecific T cell engager in multiple myeloma
Source: Sci Rep. 2022 Jun 29;12:10976. doi: 10.1038/s41598-022-14726-5 (PMC9243109; doi:10.1038/s41598-022-14726-5)
Supplement: Supplementary file 4 — Supplementary Information 4. [file 41598_2022_14726_MOESM4_ESM.docx]

**Supplementary Information for**

Quantitative systems pharmacology modeling sheds light into the dose response relationship of a trispecific T cell engager in multiple myeloma

Abrams, R.E.^1,2^, Pierre, K. ^1*^, El-Murr, N.^3^, Seung, E.^4,5^, Wu, L.^4,5^, Luna, E.^6^, Mehta, R.^6^,Li, J.^1^, Larabi, K.^3^, Ahmed, M.^7^, Pelekanou, V.^7,8^, Yang Z-Y.^4,5^, van de Velde, H.^7^, Stamatelos, S.K. ^1,9*^

*1 Sanofi, Bridgewater, 55 Corporate Dr., Bridgewater, NJ, 08807 USA
 2 Current address: Daichi Sankyo, 211 Mt. Airy Rd., Basking Ridge, NJ 07920
 3 Sanofi, 13 quai Jules Guesde 94403 Cedex,VITRY-SUR-SEINE, Vitry/Alforville France
 4 Sanofi, 270 Albany St., Cambridge, MA 02139, USA
 5 Current address: Modex Therapeutics, 22 Strathmore Road, Natick, MA 01760
 6 Sanofi,* *Orlando, FL, USA
 7 Sanofi, 50 Binney St., Cambridge, MA 02142, USA*

*8 Current Address: Bayer Pharmaceuticals, Cambridge, MA 02142, USA*
 *9 Current Address: Bayer Pharmaceuticals, PH100 Bayer Boulevard, Whippany, NJ, 07981, USA*

***Corresponding Authors:** Kamau Pierre and Spyros K. Stamatelos

**Email:**  kamau.pierre@sanofi.com and spyros.stamatelos@bayer.com

**This PDF file includes:**

Supplementary methods for experimental data

Figures S1 to S9

Tables S1 to S2

Supplementary References

Supplementary Methods

**Purification of human PBMC from buffy coats**

Human buffy coat from healthy donors were purchased from Research Blood Components, LLC. Peripheral blood mononuclear cells (PBMC) were isolated from human buffy coat by Ficoll-Paque Plus density separation with Leucosep® tubes. Red blood cells were lysed by adding 5ml Ammonium-Chloride-Potassium (ACK) Lysing Buffer for 3 to 5 minutes at room temperature. Cells were washed with PBS and resuspend at 2 x 10^6^ cells/ml in RPMI1640 culture medium with 10% FBS.

**Purification of isolated T-cells**

Untouched T cells from enriched PBMC were isolated using Pan T Cell Isolation Kit (Miltenyi Biotec). Isolation buffer was prepared with phosphate buffered saline (PBS), pH 7.2, 0.5% bovine serum albumin (BSA), and 2 mM EDTA. Procedures were followed as described in manufactory data sheets.

**Tumor cell lines and culture conditions**

The human myeloma cell line KMS-11 was obtained from the JCRB Cell Bank and the human myeloma cell line RPMI 8226 (CCL-155) was obtained from the American Type Culture Collection (ATCC). Cells were maintained in RPMI 1640 GlutaMAX Medium (ThermoFisher Scientific) supplemented with 10% heat inactivated fetal bovine serum (FBS) in a humidified incubator at 37°C and 5% CO_2_.

**Activation assay with PBMCs**

Serially diluted testing materials were added to human PBMCs purified from healthy donors at 1 x 10^6^ cells/mL with total 200 μl/well in 96-well culture plate. After incubating at 37 °C for 24 hours, the cells were stained with anti-CD3‑PE, anti-CD8‑BV421, anti-CD4‑PerCP/Cy5.5, and anti-CD69‑APC/Cy7, and analyzed by flow cytometry for measuring percentage of CD69+CD3+ human T cells. The activation activity was reported as the percentages of CD69+/ CD3+ T cells among CD3+ T cells.

**Tumor cell killing using effector CD8+ T cells.**

Tumor cell killing in the presence of effector CD8+ T cells was performed using the Calcein release method. KMS-11 and RPMI 8226 cells were first labeled with Calcein AM (ThermoFisher Scientific) for 30 minutes at 37°C, washed multiple times and then seeded in 96-well flat bottom plates (2.10^4^ cell/well) in RPMI 1640 GlutaMAX Medium supplemented with 10% FBS and Probenecid (ThermoFisher Scientific) in the presence of different concentrations of antibodies and activated CD8+ T cells (1:10 effector to target ratio). Activated CD8+ T cells (MART-1 specific) were a kind gift from Dr. Laurent Vidard (Sanofi Oncology R&D, Vitry-sur-Seine) and were activated using a mix of cytokines (Il-7, Il-15 and Il-21) and antigen presenting cells (K562 cell line) artificially expressing MART-1 and HLA-A2. The mix was then incubated for 4 hours at 37°C in a humidified incubator. Plates were then centrifuged and 100 µL of the supernatant were transferred into a black 96-well plate. Fluorescent calcein release was quantified using a fluorescence microplate reader (TECAN Spark 10M). Control wells included non-treated cells (background release of calcein), and triton-treated cells (positive control, 100% killing). Activated T cells were amplified from healthy donor (HD) PBMC. PBMC were isolated from HD buffy coats obtained from the “Etablissement Français du Sang” under French regulations regarding the use of human blood or human blood components for research purposes (contract reference: C CPSL UNT – Number: 12/EFS/131). This contract covers the informed consent for all donors.

**Cytotoxicity assay with PBMCs**

Multiple Myeloma (MM) tumor target cell lines RPMI8226 and KMS11 were labeled with PKH26 red fluorescent membrane dye following manufacturer’s instruction. Serial diluted trispecific antibody were added into U-bottom culture platecontaining 2x10^4^ cells/well pre-labeled target cells and co-cultured with 2x10^5^ cells/well human PBMCs as effector cells (E:T=10:1). After incubating at 37°C for 20-24 hours, the cells were collected and stained with violet dead cell staining dye (BV421) following manufacturer’s instruction and analyzed by flow cytometry. All data exported FCS data were analyzed with Flowjo software version 10. The target cell was gated by gating PE+ BV421- population. The percentage of cytolytic activity was calculated by the average of dead cell percentage subtracting the average of dead cell percentage from no antibody treated control group.

**Proliferation assay**

To measure the effect of the CD38xCD28xCD3 trispecific antibody on primary human T cells, the antibody was added to 96-well microtiter plates for a final concentration of 50 nM after adding primary human T cells. The plates were incubated for 1, 3 and 6 days at 37°C before cells were analyzed by flow cytometry. T cell proliferation was assessed by using flow cytometry to measure the number of T cells at various time points. Data from flow cytometric acquisition on the BD LSRFortessa was analyzed using FlowJo™10.4.2 software.

**MIMIC assay**

All PBMC collections and use in VaxDesign, Orlando are according to IRB permission (protocol number: 0906009) through Advarra, 6940 Columbia Gateway Dr, Suite 110, Columbia, MD 21046 (formerly Chesapeake Research Review).

**Leucocytes and plasma preparation**

Fresh leukocytes (RBC-depleted whole blood cells) were acquired from blood donations from normal healthy donors who provide informed consent and were enrolled in the Sanofi VaxDesign donor program (Chesapeake Research Review, Inc., Columbia, MD., protocol 0906009). The blood components were isolated according to the procedure, **GDMS_597366**_Adult Whole Blood Processing Granulocyte Isolation. Briefly, from each volunteer’s sample, whole blood leukocytes and their corresponding autologous platelet-poor plasma were separated by centrifugation at 2100 rpm. The separated plasma was spun down and cleared through a 0.2-μm filter (polyetherslfone; Nalgene, Rochester, NY) to remove platelets and platelet particles (PPP). To remove red blood cells, the leukocyte pellets were mixed with a sterile solution of 5% (w/v) dextran (Sigma, St Louis, MO) to allow sedimentation of the erythrocytes. Thereafter, supernatants were collected and washed two times with DPBS (Lonza). Then, white cells were counted using trypan blue exclusion staining.

**MIMIC CRA procedure**

The procedure for the automated MIMIC VPTE CRA is described in detail in the Standard Operating Procedure, **GDMS_597285**. Briefly, on day 0, 96-well plates were coated with a collagen solution. One day later, an endothelial cell line was seeded onto the collagen cushion in a media-serum solution. After growth until confluency (about 4 days), the media was exchanged to a non-serum media. One day later, fresh reconstituted leucocytes were applied to the construct along with test agents. After 20-24 hours, culture supernatants were collected and analyzed for cytokine/chemokine production using multiplex arrays.

**Evaluation of cytokine/chemokine secretion using multiplex array**

MIMIC® CRA culture supernatants were analyzed using EMD Millipore’s MILLIPLEX® MAP Human Cytokine / Chemokine Magnetic Bead Panel. This kit was used for the quantification of the following human cytokines and chemokines: IFN-γ, IL-1β, IL-2, IL-4, IL-5, IL-6, IL-10, MCP-1, MIP-1β and TNF-α. The manufacturer’s protocol was followed, as prescribed, with some minor modifications that are described in procedure **GDMS_599533,** Using the Bio-Plex^TM^/ Luminex Systems to Assess Cytokine and Chemokine Levels. Culture supernatant samples were diluted 1:2 to 1:20 in serum-free culture media as a starting point for the bioplex analysis. Depending on the concentration of cytokines/chemokines in the samples, it was in some cases necessary to adjust the supernatant sample dilution above or below these starting dilution values. The kit includes human cytokine quality controls for each analyte; these samples were diluted 2- and 4-fold and run on each plate. The kit also includes human cytokine/chemokine standards for each analyte; an 8-point dilution curve, representing concentrations from 10,000 – 4.57 pg/mL, was generated for each analyte using these standards.

For run acceptance criteria, LLOQ and ULOQ for each analyte was established based on the percent recovery (Observed/Expected*100) of each point against the 5-parameter logistic (5PL) curve fit of the standard values. A recovery range of 80% - 120% was considered acceptable, such that values falling within this range defined the lower and upper bounds of the standard curve. The raw data file was reviewed for bead counts; a data point was considered valid when a minimum of 35 beads were counted per region.

**Data analysis and graphical plotting**

All data was exported into excel databases. Out-of-range high (>OOR) values (values greater than the highest point of the curve) were removed from the table. Out-of-range low (<OOR) values were replaced with 1/2 the LLOQ. Data were exported to GraphPad Prism for statistical analyses and graph preparation.

**Statistical analysis**

To estimate EC20 and EC50 induced by the trispecific compound, the biostatistics team wrote a SAS macro that reads in these excel files (one by one), extracts the necessary information, and stores them in a subsequent dataset. Consequently, this SAS macro compiles all 28 datasets, merges them into one dataset, and exports it as an xlsx format under the name, 00Combined_data.xlsx, for further statistical analysis. Data processing was conducted using statistical software, SAS 9.4. Effective concentration 50% (EC50), including 95%CI as the measure of the accuracy of each estimate per donor and per cytokine, were calculated from this dataset. Then, the geometric means of EC50 values among all donors (whose dose-response curve could be fitted), as well as, the geometric STD of the geometric mean, as a measure of variability among different donors, were estimated per treatment per cytokine. Geometric means and 95% confidence intervals (CIs) were computed after removing those donors having out of range relative EC50 estimates. The data was displayed as fitted curves alone along with the responses per donor when convergence was achieved. For a more detailed explanation of this analysis, please refer to the associated biostatistical report.

**
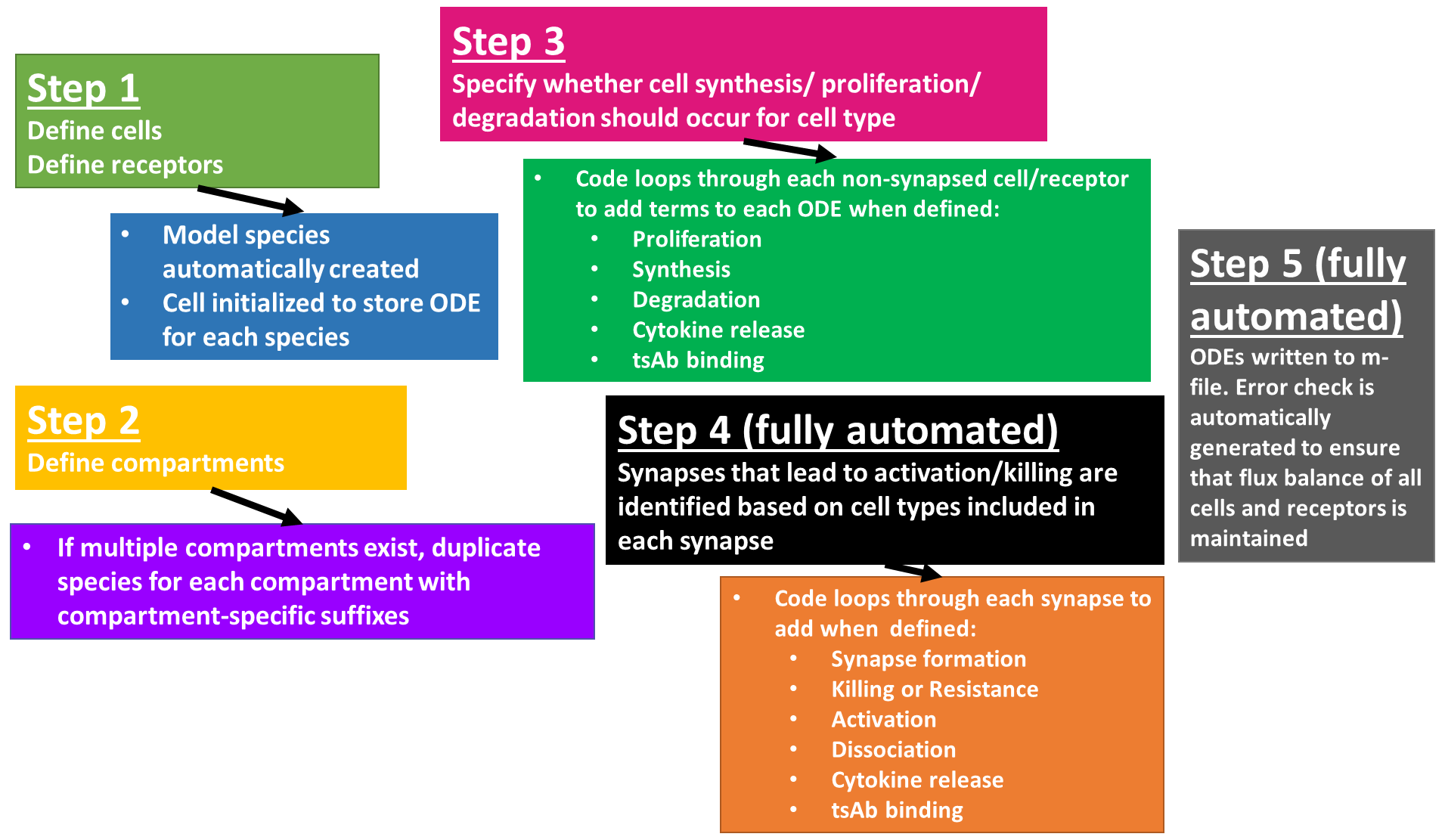
**

**Figure S1. Rule-based model generation procedure.** Workflow of rule-based model generation showing what must be specified and what is automated. Initial step describes what user must specify/is specified and following box specifies result in model.


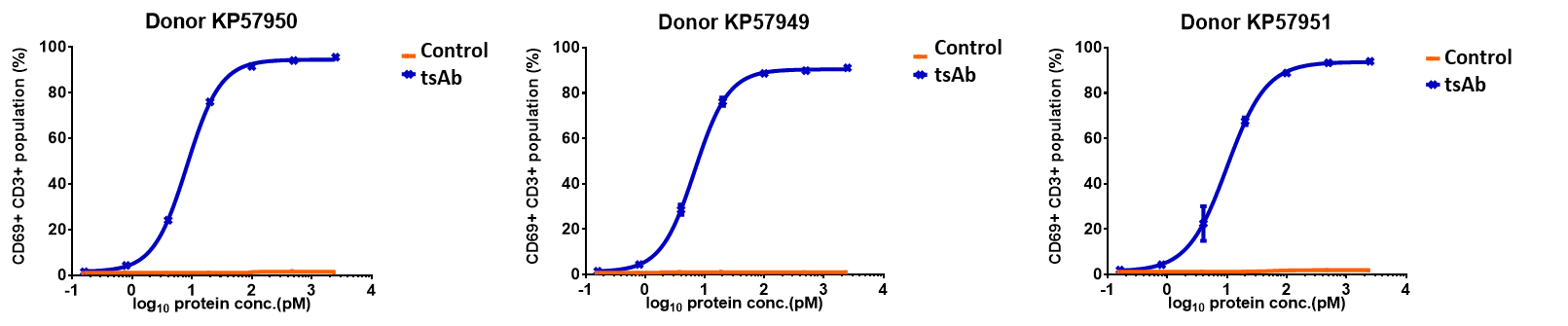


**Fig. S2. In vitro human primary T cell activation by CD3-CD38-CD28 trispecific antibody** T-cell activation assay was performed on samples from three donors. Results are compared to control.


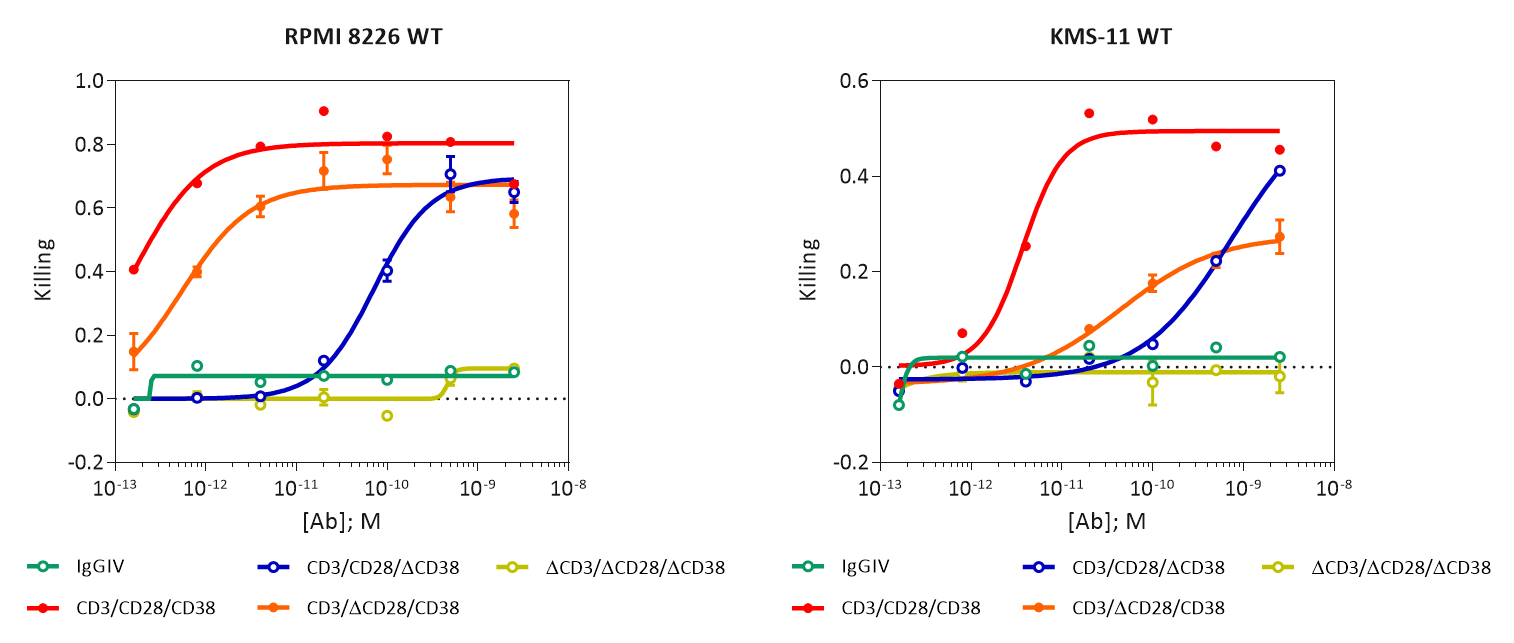


**Fig. S3. Cytotoxicity assay with effector T-cells** Cytotoxicity assay was performed by incubating effector T-cells with two different tumor cell lines, RPMI 8826 and KMS-11. Trispecific experiments were compared to different bispecific or control constructs.


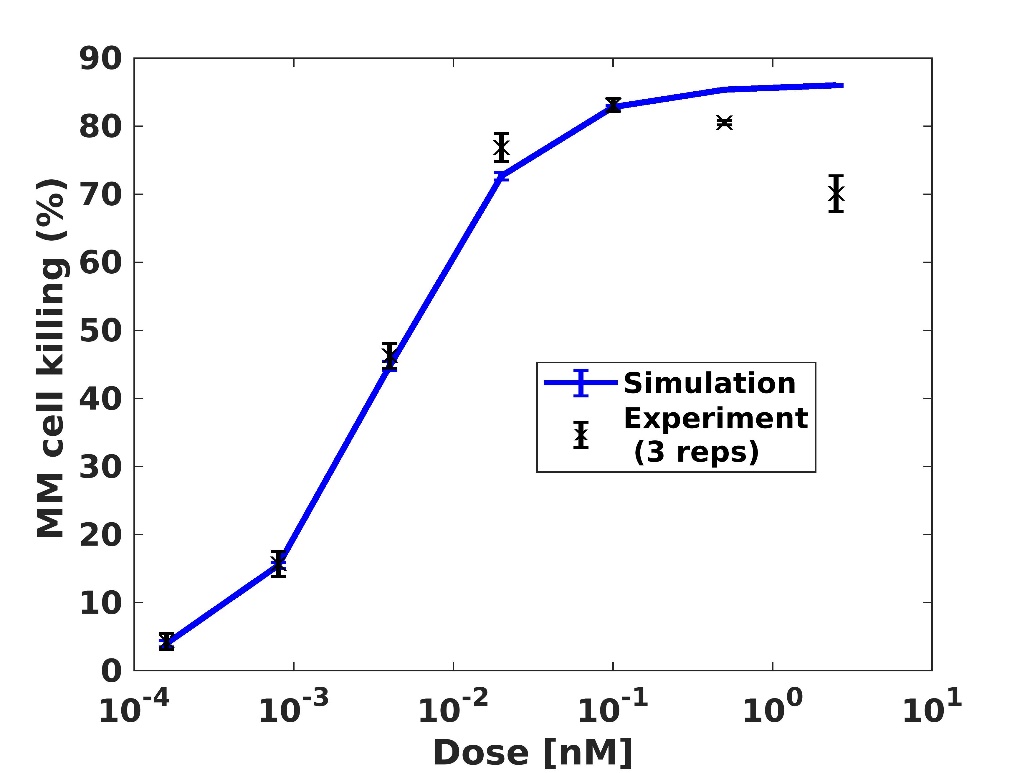


**Fig. S4.** Calibration of the model to cytotoxicity data from KMS-11 cell line
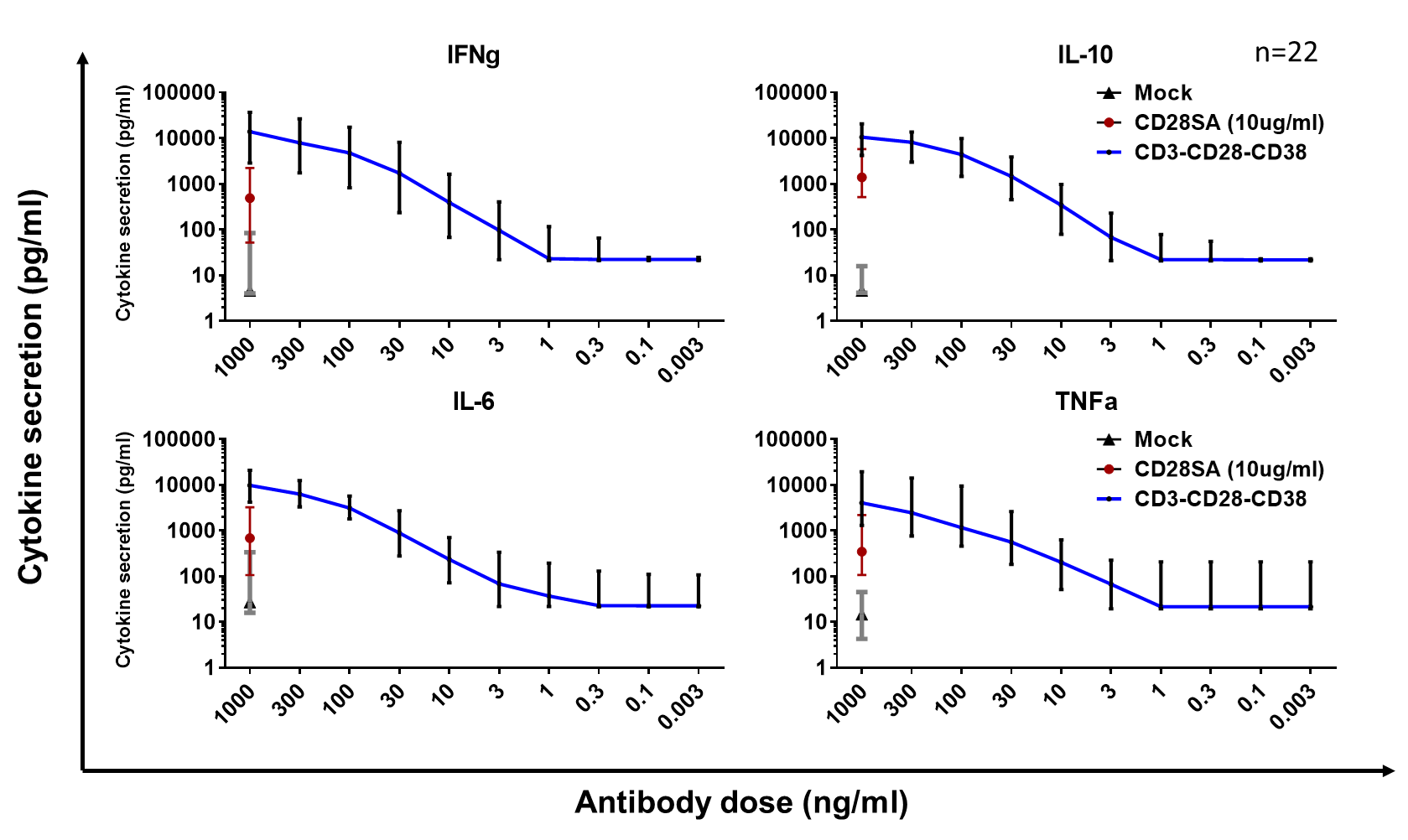


**Fig. S5. Strong dose dependent secretion of T cell-derived cytokines in the MIMIC culture supernatant after 20hr treatment with anti-CD3-CD28-CD38 antibody.** MIMIC CRA cultures were treated with the indicated compounds/doses for 20-22hr and then the culture supernatants were harvested for cytokine/chemokine milliplex analysis. Lines represent median with range of secretion (pg/ml) for IFNg, IL-10, IL-6 and TNFa. CD28 super agonistic antibody (10ug/ml) was employed as assay control. N = 22 healthy adult donors.


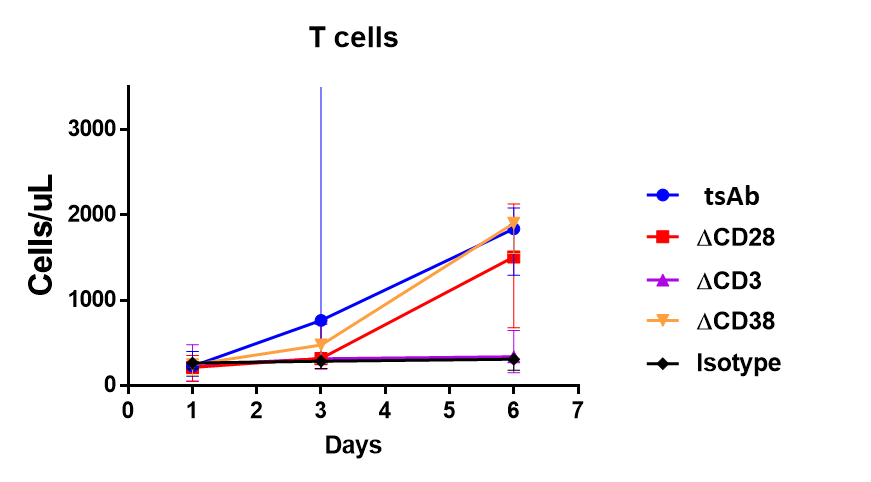


**Fig. S6. Proliferation of T cells after stimulation with trispecific and its knockout variants after 1 to 6 days of incubation at 50 nM (median ± 95% CI of 4 donors)**


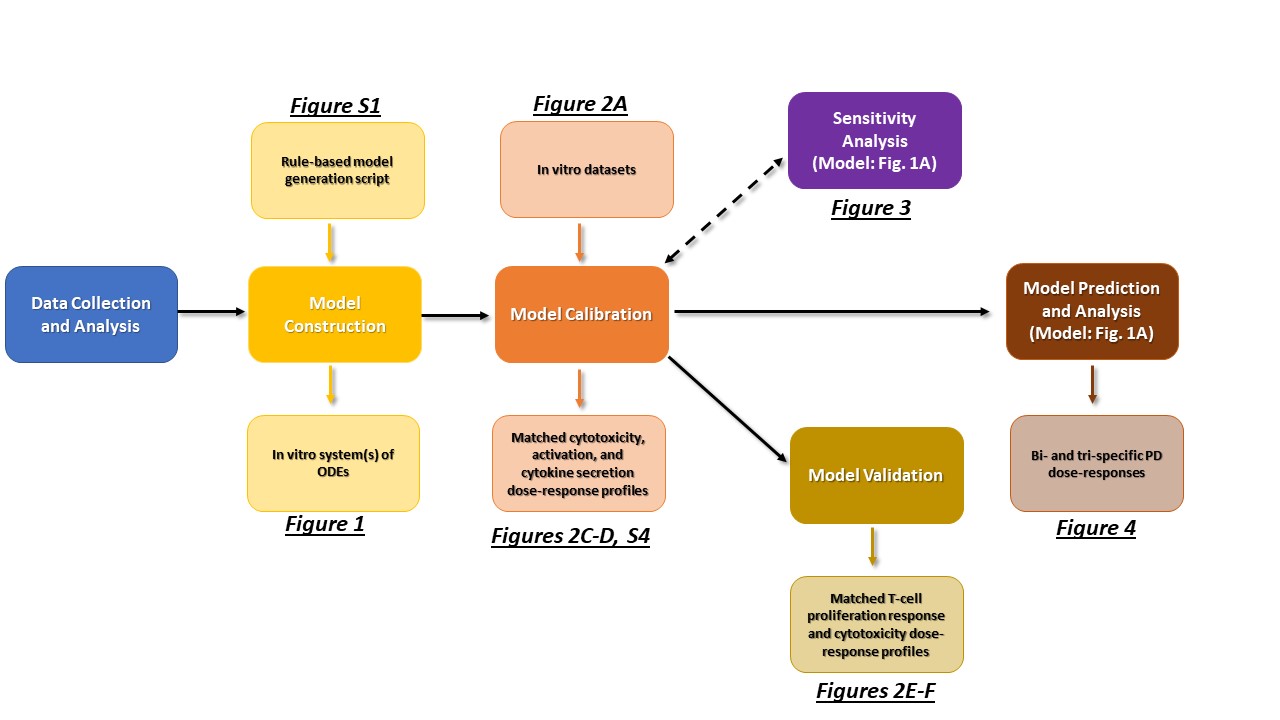


**Fig. S7. Model development and assessment workflow.** Collected datasets were analyzed and used in inform model structure with the final model generated via the rule-based script. In vitro datasets were utilized to calibrate the model whereby select parameters were optimized to ensure goodness of fit with the experimental data. Sensitivity analysis was used to both identify pertinent parameters for optimization, informing the model calibration process, but to also assess the impact of parameter variation on model output. Simulation results from the finalized model were assessed and the predictive capabilities of the model were confirmed by the model validation process. Colored arrows correspond to inputs and outputs whereas black arrows connect processes.


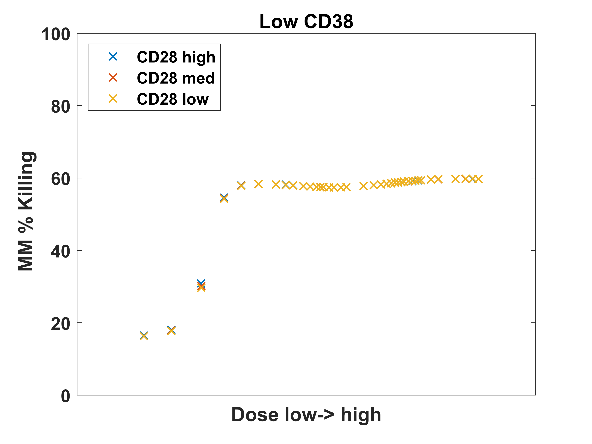

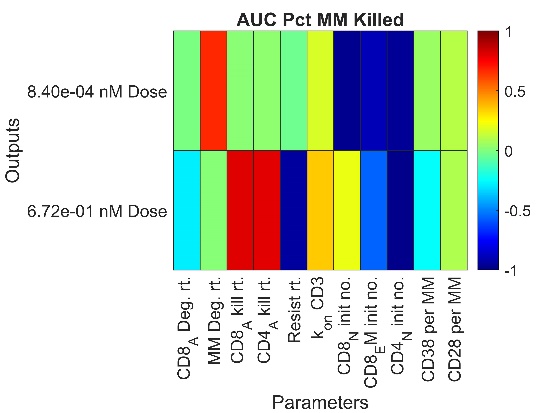


**Fig. S8. Impact of MM CD28 expression on efficacy.** Variation of CD28 expression levels across reported values (low, median and high) has minimal impact on tumor cell killing across all doses explored (left). The lack of sensitivity of MM cell killing to CD28 expression on tumor cells is confirmed via sensitivity analysis (right) with a near zero correlation of this parameter (CD28 per MM) to the percent of myeloma cells killed.


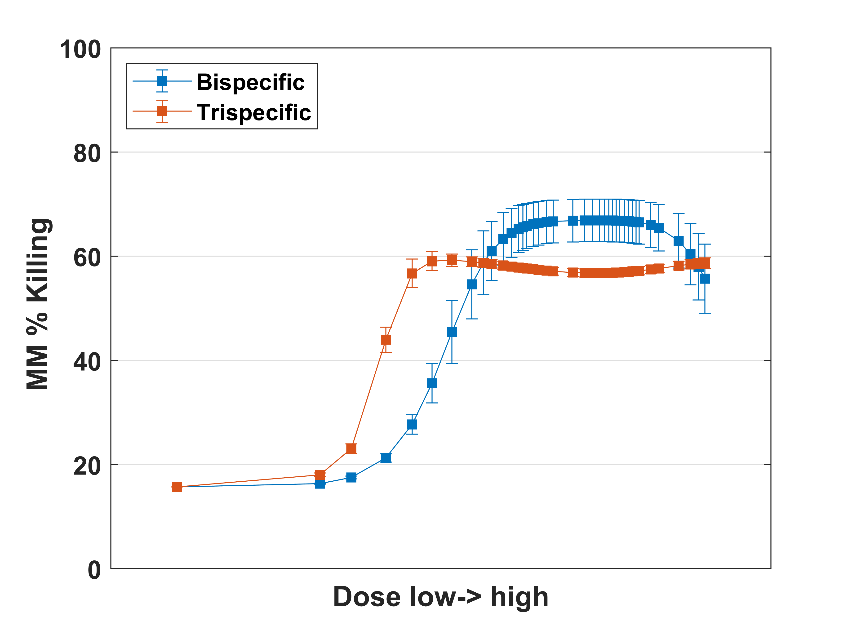


**Fig. S9. Trispecific is still superior to bispecific antibody at lower doses despite downregulation of CD28 expression on T cells.** Reducing the number of CD28 sites on T cells does not drastically impact the efficacy of the trispecific antibody as a similar dose-dependent response is predicted when compared to the nominal CD28 expression case (Figure 4C).

**Table S1. Rationale and literature basis for key model assumptions**


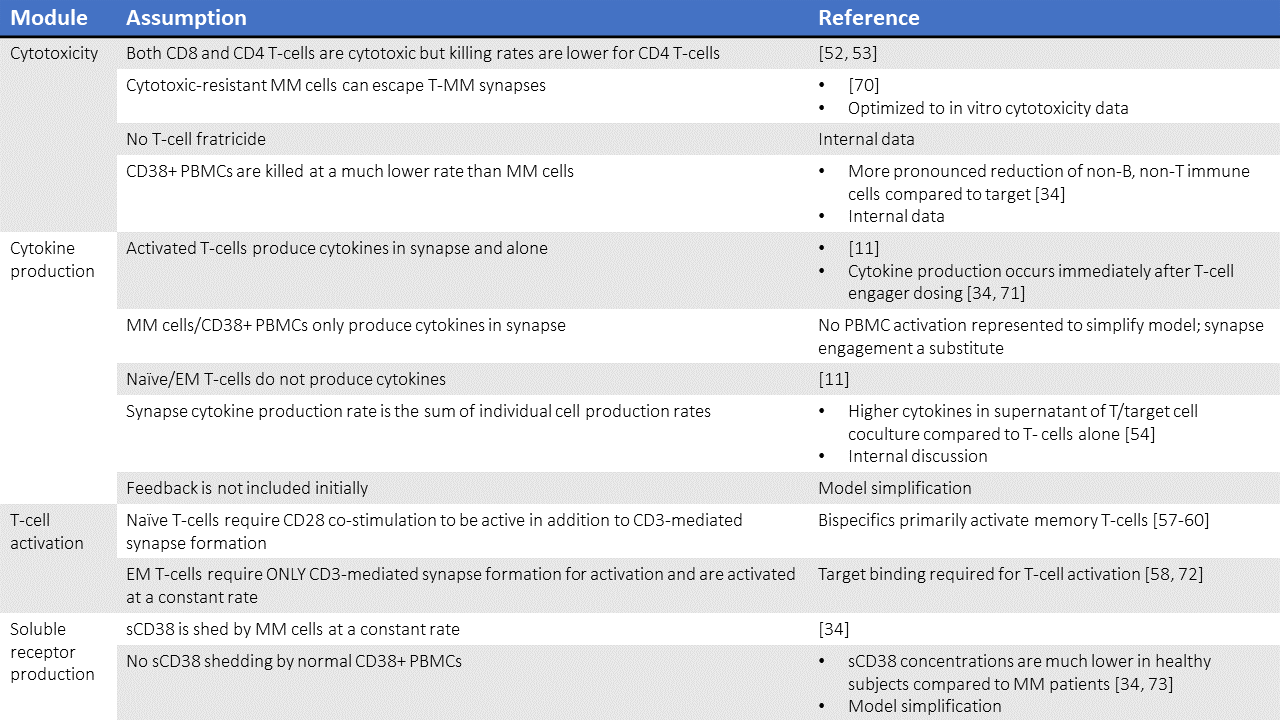


**Table S2. Parameters and initial values used in the in vitro model**

| Parameter | Description | Value | Unit | Source for value or range used in calibration | Calibrated against |
| --- | --- | --- | --- | --- | --- |
| ksyn_CD8_N | Synthesis of naïve CD8+ T-cells | 0 | Cells/hr | Assume no synthesis in vitro | N.A. |
| ksyn_CD8_EM | Synthesis of effector memory CD8+ T-cells | 0 | Cells/hr | Assume no synthesis in vitro | N.A. |
| ksyn_CD4_N | Synthesis of naïve CD4+ T-cells | 0 | Cells/hr | Assume no synthesis in vitro | N.A. |
| ksyn_CD4_EM | Synthesis of effector memory CD4+ T-cells | 0 | Cells/hr | Assume no synthesis in vitro | N.A. |
| kdeg_CD8_N | *In vitro* apoptotic rate of naïve CD8+ T-cells | 0.0064 | 1/hr | [74] | N.A. |
| kdeg_CD8_EM | *In vitro* apoptotic rate of effector memory CD8+ T-cells | 0.0076 | 1/hr | [74] | N.A. |
| kdeg_CD8_A | *In vitro* apoptotic rate of active CD8+ T-cells | 0.051 | 1/hr | [75] | MIMIC assay |
| kdeg_CD4_N | *In vitro* apoptotic rate of naïve CD4+ T-cells | 0.0085 | 1/hr | [76] | N.A. |
| kdeg_CD4_EM | *In vitro* apoptotic rate of effector memory CD4+ T-cells | 0.0076 | 1/hr | [76] | N.A. |
| kdeg_CD4_A | *In vitro* apoptotic rate of active CD4+ T-cells | 0.01 | 1/hr | [75] | MIMIC assay |
| kdeg_MM | *In vitro* apoptotic rate of multiply myeloma cells | 0.0025 | 1/hr | [77] | N.A. |
| kdeg_TRGT | *In vitro* apoptotic rate of CD38+ PBMCs | 0.0036 | 1/hr | [78] | MIMIC assay |
| kpr_CD8 | *In vitro* proliferation rate of active CD8+ T-cells | 0.0452 | 1/hr | [41] | Activation assay |
| kpr_CD4 | *In vitro* proliferation rate of active CD4+ T-cells | 0.0099 | 1/hr | [41] | Activation assay |
| kpr_MM | *In vitro* proliferation rate of multiple myeloma cells | 1.00e-4 | 1/hr | [77] | Cytotoxicity assay |
| kpr_TRGT | *In vitro* proliferation rate of CD38+ PBMCs | 0 | 1/hr | Assume no synthesis in vitro | N.A. |
| C_M_MM | *In vitro* MM tumor carrying capacity | 1.00e6 | Cells | [77] | Cytotoxicity assay |
| kkillMM_CD8 | Tumor killing rate of CD8+ T-cells | 64.2663 | 1/hr | Calibrated | Cytotoxicity assay |
| kkillMM_CD4 | Tumor killing rate of CD4+ T-cells | 6.4266 | 1/hr | Assume 1 order of magnitude less than CD8 killing | N.A. |
| kmut_SYN | Formation rate of tumor resistance to killing | 14.2651 | 1/hr | Calibrated | Cytotoxicity assay |
| kkillTRGT_CD8 | CD38+ PBMC killing rate of CD8+ T-cells | 1.17e-4 | 1/hr | Calibrated | MIMIC assay |
| kkillTRGT_CD4 | CD38+ PBMC killing rate of CD4+ T-cells | 3.71e-6 | 1/hr | Calibrated | MIMIC assay |
| kprod_CD8_A_IFNg | CD8+ T-cell production rate of IFNγ | 1.56e3 | Molecules/cell/hr | Calibrated | MIMIC assay |
| kprod_CD4_A_IFNg | CD4+ T-cell production rate of IFNγ | 1.32e4 | Molecules/cell/hr | Calibrated | MIMIC assay |
| kprod_TRGT_IFNg | CD38+ PBMC production rate of IFNγ | 0 | Molecules/cell/hr | Assume PBMCs only produce IL-6 and IL-10 | N.A. |
| kprod_CD8_A_TNFa | CD8+ T-cell production rate of TNFα | 147.39 | Molecules/cell/hr | Calibrated | MIMIC assay |
| kprod_CD4_A_TNFa | CD4+ T-cell production rate of TNFα | 1.94e3 | Molecules/cell/hr | Calibrated | MIMIC assay |
| kprod_TRGT_TNFa | CD38+ PBMC production rate of TNFα | 0 | Molecules/cell/hr | Assume PBMCs only produce IL-6 and IL-10 | N.A. |
| kprod_CD8_A_IL6 | CD8+ T-cell production rate of IL-6 | 896.169 | Molecules/cell/hr | Calibrated | MIMIC assay |
| kprod_CD4_A_IL6 | CD4+ T-cell production rate of IL-6 | 566.361 | Molecules/cell/hr | Calibrated | MIMIC assay |
| kprod_TRGT_IL6 | CD38+ PBMC production rate of IL-6 | 3.07e4 | Molecules/cell/hr | Calibrated | MIMIC assay |
| kprod_CD8_A_IL10 | CD8+ T-cell production rate of IL-10 | 0 | Molecules/cell/hr | Assume T-cells do not produce IL-10 | N.A. |
| kprod_CD4_A_IL10 | CD4+ T-cell production rate of IL-10 | 0 | Molecules/cell/hr | Assume T-cells do not produce IL-10 | N.A. |
| kprod_TRGT_IL10 | CD38+ PBMC production rate of IL-10 | 4.42e4 | Molecules/cell/hr | Calibrated | MIMIC assay |
| kprod_MM_IFNg/TNFa/IL6/IL10 | MM cell production rate of IFNγ /TNFα/IL-6/IL-10 |  | Molecules/cell/hr | Assume same as TRGT rates | N.A. |
| kdeg_IFNg/TNFa/IL6/IL10 | Degradation rate of IFNγ /TNFα/IL-6/IL-10 | 0 | 1/hr | Assume no significant degradation in vitro | N.A. |
| kshedMM_s38 | Shedding rate of soluble CD38 from MM cells | 0.005 | Molecules/cell/hr | Calibrated | Cytotoxicity assay |
| kshedTRGT_s38 | Shedding rate of soluble CD38 from CD38+ PBMCs | 0 | Molecules/cell/hr | Assume only significant shedding is from MM cells | N.A. |
| kdeg_sCD38 | Degradation rate of soluble CD38 | 0 | 1/hr | Assume no significant degradation in vitro | N.A. |
| kact_N | Activation rate of naïve T-cells | 101 | 1/hr | Calibrated | Activation assay |
| kact_EM | Activation rate of effector memory T-cells | 2.14 | 1/hr | Calibrated | Activation assay |
| EC50_CD3 | EC50 of fraction of CD3 bound by drug needed for naïve T-cell activation | 0.0169 | ~ | Calibrated | Activation assay |
| EC50_CD28 | EC50 of fraction of CD28 bound by drug needed for naïve T-cell activation | 0.0137 | ~ | Calibrated | Activation assay |
| E | Error factor in naïve cell activation to prevent division by 0 | 1 | Molecules | Set to small error value | N.A. |
| CD3per_CD4 (A, EM, N) | CD3 antigen density on CD4+ T-cells | 124000 | Molecules/cell | [62] | N.A. |
| CD3per_CD8 (A, EM, N) | CD3 antigen density on CD8+ T-cells | 124000 | Molecules/cell | [62] | N.A. |
| CD28per_CD4 (A, EM, N) | CD28 antigen density on CD4+ T-cells | 19000 | Molecules/cell | [51] | N.A. |
| CD28per_CD8 (A, EM, N) | CD28 antigen density on CD8+ T-cells | 12500 | Molecules/cell | [51] | N.A. |
| CD28per_MM | CD28 antigen density on MM cells | 50000 | Molecules/cell | Average of CD28 densities on KMS-11 and RPMI-8226 cell lines | N.A. |
| CD38per_MM | CD38 antigen density on MM cells | 23000 | Molecules/cell | [50, 66] | N.A. |
| CD28per_TRGT | CD28 antigen density on CD38+ PBMCs | 0 | Molecules/cell | Assume no significant CD28 expression on CD38+ PBMCs | N.A. |
| CD38per_TRGT | CD38 antigen density on CD38+ PBMCs | 3.32e3 | Molecules/cell | Average of B-cell, Monocytes, NK cells. [50, 64-66]  Daratumumab downregulation factor applied. [50] | N.A. |
| Vol | In vitro well volume | 2.00e-4 | L | Volume of internal in vitro experiments | N.A. |
| kon_CD3 | Binding rate of drug to CD3 antigen | 6.7e-13 |  | Internal data [9] | N.A. |
| koff_CD3 | Dissociation rate of drug from CD3 antigen | 1.6524 |  | Internal data [9] | N.A. |
| kon_CD28 | Binding rate of drug to CD28 antigen | 2.8e-12 |  | Internal data [9] | N.A. |
| koff_CD28 | Dissociation rate of drug from CD28 antigen | 0.7344 |  | Internal data [9] | N.A. |
| kon_CD38 | Binding rate of drug to CD38 antigen | 1.36e-11 |  | Internal data [9] | N.A. |
| koff_CD38 | Dissociation rate of drug from CD38 antigen | 7.2 |  | Internal data [9] | N.A. |
| kcoll | Collision factor / Propensity of collision between two cells | 82.7 |  | Calibrated | Activation assay |
| Br_perS | Number of bridges between receptors per synapse | 14.2 |  | Calibrated | Activation assay |
| kDis | Dissociation rate of synapses | 2.68e-4 |  | Calibrated | Activation assay |
| CD8_N_0 | Scaling factor for naïve CD8+ T-cells used in synapse formation term | 20274 |  | Set to initial cell number | N.A. |
| CD8_EM_0 | Scaling factor for effector memory CD8+ T-cells used in synapse formation term | 20274 |  | Set to initial cell number | N.A. |
| CD8_A_0 | Scaling factor for active CD8+ T-cells used in synapse formation term | 20274 |  | Set to initial cell number | N.A. |
| CD4_N_0 | Scaling factor for naïve CD4+ T-cells used in synapse formation term | 10488 |  | Set to initial cell number | N.A. |
| CD4_EM_0 | Scaling factor for effector memory CD4+ T-cells used in synapse formation term | 10488 |  | Set to initial cell number | N.A. |
| CD4_A_0 | Scaling factor for active CD4+ T-cells used in synapse formation term | 10488 |  | Set to initial cell number | N.A. |
| TRGT_0 | Scaling factor for CD38+ PBMCs used in synapse formation term | 4960000 |  | Set to initial cell number | N.A. |
| MM_0 | Scaling factor for MM cells used in synapse formation term | 2540800 |  | Set to initial cell number | N.A. |
| Initial Value | **Description** | **Value** | **Unit** | **Source** | **Calibrated against** |
| CD8_N | CD8+ Naive T-cells | 1.55e4 | Cells | [39, 67] | N.A. |
| CD8_EM | CD8+ Effector Memory T-cells | 8.12e3 | Cells | [39, 67] | MIMIC assay |
| CD8_A | CD8+ Active T-cells | 2.36e2 | Cells | [39, 67] | MIMIC assay |
| CD4_N | CD4+ Naive T-cells | 1.20e4 | Cells | [39, 67] | N.A. |
| CD4_EM | CD4+ Effector Memory T-cells | 3.11e3 | Cells | [39, 67] | MIMIC assay |
| CD4_A | CD4+ Active T-cells | 2.11e2 | Cells | [39, 67] | MIMIC assay |
| MM | Multiple Myeloma cells | 3.92e3 | Cells | [79] | N.A. |
| TRGT | CD38+ PBMCs | 2.90e4 | Cells | [39, 67] | N.A. |
| sCD38 | Soluble CD38 | 4.96e6 | Molecules | [80] | N.A. |
| *Note: Model was constructed in terms of cell/receptor numbers rather than concentrations | | | | | |

**Supplementary References**

9 Wu, L. *et al.* Trispecific antibodies enhance the therapeutic efficacy of tumor-directed T cells through T cell receptor co-stimulation. *Nature Cancer* **1**, 86-98, doi:10.1038/s43018-019-0004-z (2020).

11 Pennock, N. D. *et al.* T cell responses: naive to memory and everything in between. *Adv Physiol Educ* **37**, 273-283, doi:10.1152/advan.00066.2013 (2013).

34 de Zafra, C. L. Z. *et al.* Targeting Multiple Myeloma with AMG 424, a Novel Anti-CD38/CD3 Bispecific T Cell-Recruiting Antibody Optimized for Cytotoxicity and Cytokine Release. *Clinical Cancer Research*, clincanres. 2752.2018 (2019).

39 Zelle-Rieser, C. *et al.* T cells in multiple myeloma display features of exhaustion and senescence at the tumor site. *Journal of hematology & oncology* **9**, 116 (2016).

41 Li, Y. & Kurlander, R. J. Comparison of anti-CD3 and anti-CD28-coated beads with soluble anti-CD3 for expanding human T cells: differing impact on CD8 T cell phenotype and responsiveness to restimulation. *Journal of translational medicine* **8**, 104 (2010).

50 Krejcik, J. *et al.* Monocytes and Granulocytes Reduce CD38 Expression Levels on Myeloma Cells in Patients Treated with Daratumumab. *Clinical cancer research : an official journal of the American Association for Cancer Research* **23**, 7498-7511, doi:10.1158/1078-0432.Ccr-17-2027 (2017).

51 Bryl, E. *et al.* Modulation of CD28 expression with anti–tumor necrosis factor α therapy in rheumatoid arthritis. *Arthritis & Rheumatism: Official Journal of the American College of Rheumatology* **52**, 2996-3003 (2005).

52 Haagen, I.-A. *et al.* Unprimed CD4+ and CD8+ T cells can be rapidly activated by a CD3× CD19 bispecific antibody to proliferate and become cytotoxic. *Cancer Immunology, Immunotherapy* **39**, 391-396 (1994).

53 Mack, M., Gruber, R., Schmidt, S., Riethmüller, G. & Kufer, P. Biologic properties of a bispecific single-chain antibody directed against 17-1A (EpCAM) and CD3: tumor cell-dependent T cell stimulation and cytotoxic activity. *the Journal of Immunology* **158**, 3965-3970 (1997).

54 Giuliani, N. *et al.* Human myeloma cells stimulate the receptor activator of nuclear factor-κB ligand (RANKL) in T lymphocytes: a potential role in multiple myeloma bone disease. *Blood, The Journal of the American Society of Hematology* **100**, 4615-4621 (2002).

57 Kufer, P. *et al.* Minimal costimulatory requirements for T cell priming and TH1 differentiation: activation of naive human T lymphocytes by tumor cells armed with bifunctional antibody constructs. *Cancer Immunity Archive* **1** (2001).

58 Krupka, C. *et al.* CD33 target validation and sustained depletion of AML blasts in long-term cultures by the bispecific T-cell–engaging antibody AMG 330. *Blood* **123**, 356-365 (2014).

59 Klinger, M. *et al.* Immunopharmacologic response of patients with B-lineage acute lymphoblastic leukemia to continuous infusion of T cell–engaging CD19/CD3-bispecific BiTE antibody blinatumomab. *Blood, The Journal of the American Society of Hematology* **119**, 6226-6233 (2012).

60 Bargou, R. *et al.* Tumor regression in cancer patients by very low doses of a T cell-engaging antibody. *Science (New York, N.Y.)* **321**, 974-977, doi:10.1126/science.1158545 (2008).

62 Ginaldi, L. *et al.* Differential expression of CD3 and CD7 in T‐cell malignancies: a quantitative study by flow cytometry. *British journal of haematology* **93**, 921-927 (1996).

64 Long, B. R. et al. Conferral of enhanced natural killer cell function by KIR3DS1 in early human immunodeficiency virus type 1 infection. Journal of virology 82, 4785-4792 (2008).

65 Almeida, M., Cordero, M., Almeida, J. & Orfao, A. Relationship between CD38 expression on peripheral blood T‐cells and monocytes, and response to antiretroviral therapy: A one‐year longitudinal study of a cohort of chronically infected ART‐naive HIV‐1+ patients. Cytometry Part B: Clinical Cytometry: The Journal of the International Society for Analytical Cytology 72, 22-33 (2007).

66 Kriegsmann, K. et al. Quantification of number of CD38 sites on bone marrow plasma cells in patients with light chain amyloidosis and smoldering multiple myeloma. Cytometry part B: Clinical cytometry 94, 767-776 (2018).

67 Stemcell Technologies. Frequency of Cell Types in Human Peripheral Blood. https://www.stemcell.com/media/files/wallchart/WA10006-Frequencies_Cell_Types_Human_Peripheral_Blood.pdf. Accessed June 2019., Revised 2019).

68 Vanlier, J., Tiemann, C. A., Hilbers, P. A. & van Riel, N. A. An integrated strategy for prediction uncertainty analysis. Bioinformatics 28, 1130-1135 (2012).

69 Marino, S., Hogue, I. B., Ray, C. J. & Kirschner, D. E. A methodology for performing global uncertainty and sensitivity analysis in systems biology. Journal of theoretical biology 254, 178-196 (2008).

70 Díaz, L. R. et al. Imbalance of immunological synapse-kinapse states reflects tumor escape to immunity in glioblastoma. JCI Insight 3, doi:10.1172/jci.insight.120757 (2018).

71 Huehls, A. M., Coupet, T. A. & Sentman, C. L. Bispecific T‐cell engagers for cancer immunotherapy. Immunology and cell biology 93, 290-296 (2015).

72 Al-Hussaini, M. et al. Targeting CD123 in acute myeloid leukemia using a T-cell–directed dual-affinity retargeting platform. Blood 127, 122-131 (2016).

73 Funaro, A. et al. Identification and characterization of an active soluble form of human CD38 in normal and pathological fluids. International immunology 8, 1643-1650 (1996).

74 Wallace, D. L. et al. Prolonged exposure of naïve CD8+ T cells to interleukin‐7 or interleukin‐15 stimulates proliferation without differentiation or loss of telomere length. Immunology 119, 243-253 (2006).

75 Brunetti, M. et al. Spontaneous and glucocorticoid-induced apoptosis in human mature T lymphocytes. (1995).

76 Jaleco, S. et al. Homeostasis of naive and memory CD4+ T cells: IL-2 and IL-7 differentially regulate the balance between proliferation and Fas-mediated apoptosis. The Journal of Immunology 171, 61-68 (2003).

77 Zlei, M. et al. Characterization of in vitro growth of multiple myeloma cells. Experimental hematology 35, 1550-1561 (2007).

78 Saito, T. et al. Spontaneous ex vivo apoptosis of peripheral blood mononuclear cells in patients with head and neck cancer. Clinical Cancer Research 5, 1263-1273 (1999).

79 Rawstron, A. C. et al. Circulating plasma cells in multiple myeloma: characterization and correlation with disease stage. British journal of haematology 97, 46-55 (1997).

80 Li, T. et al. Nanobody-based dual epitopes protein identification (DepID) assay for measuring soluble CD38 in plasma of multiple myeloma patients. Analytica chimica acta 1029, 65-71 (2018).
